# Precision Assessment of Real-World Associations Between Stress and Sleep Duration Using Actigraphy Data Collected Continuously for an Academic Year: Individual-Level Modeling Study

**DOI:** 10.2196/53441

**Published:** 2024-04-30

**Authors:** Constanza M Vidal Bustamante, Garth Coombs III, Habiballah Rahimi-Eichi, Patrick Mair, Jukka-Pekka Onnela, Justin T Baker, Randy L Buckner

**Affiliations:** 1 Department of Psychology Harvard University Cambridge, MA United States; 2 Center for Brain Science Harvard University Cambridge, MA United States; 3 Institute for Technology in Psychiatry McLean Hospital Belmont, MA United States; 4 Department of Psychiatry Harvard Medical School Boston, MA United States; 5 Department of Biostatistics Harvard University Boston, MA United States; 6 Athinoula A Martinos Center for Biomedical Imaging Massachusetts General Hospital Charlestown, MA United States

**Keywords:** deep phenotyping, individualized models, intensive longitudinal data, sleep, stress, actigraphy, accelerometer, wearable, mobile phone, digital health

## Abstract

**Background:**

Heightened stress and insufficient sleep are common in the transition to college, often co-occur, and have both been linked to negative health outcomes. A challenge concerns disentangling whether perceived stress precedes or succeeds changes in sleep. These day-to-day associations may vary across individuals, but short study periods and group-level analyses in prior research may have obscured person-specific phenotypes.

**Objective:**

This study aims to obtain stable estimates of lead-lag associations between perceived stress and objective sleep duration in the individual, unbiased by the group, by developing an individual-level linear model that can leverage intensive longitudinal data while remaining parsimonious.

**Methods:**

In total, 55 college students (n=6, 11% second-year students and n=49, 89% first-year students) volunteered to provide daily self-reports of perceived stress via a smartphone app and wore an actigraphy wristband for the estimation of daily sleep duration continuously throughout the academic year (median usable daily observations per participant: 178, IQR 65.5). The individual-level linear model, developed in a Bayesian framework, included the predictor and outcome of interest and a covariate for the day of the week to account for weekly patterns. We validated the model on the cohort of second-year students (n=6, used as a pilot sample) by applying it to variables expected to correlate positively within individuals: objective sleep duration and self-reported sleep quality. The model was then applied to the fully independent target sample of first-year students (n=49) for the examination of bidirectional associations between daily stress levels and sleep duration.

**Results:**

Proof-of-concept analyses captured expected associations between objective sleep duration and subjective sleep quality in every pilot participant. Target analyses revealed negative associations between sleep duration and perceived stress in most of the participants (45/49, 92%), but their temporal association varied. Of the 49 participants, 19 (39%) showed a significant association (probability of direction>0.975): 8 (16%) showed elevated stress in the day associated with shorter sleep later that night, 5 (10%) showed shorter sleep associated with elevated stress the next day, and 6 (12%) showed both directions of association. Of note, when analyzed using a group-based multilevel model, individual estimates were systematically attenuated, and some even reversed sign.

**Conclusions:**

The dynamic interplay of stress and sleep in daily life is likely person specific. Paired with intensive longitudinal data, our individual-level linear model provides a precision framework for the estimation of stable real-world behavioral and psychological dynamics and may support the personalized prioritization of intervention targets for health and well-being.

## Introduction

### Background

The transition to college is often accompanied by elevated stress and insufficient sleep [1-3], both of which have been found to impact daily functioning and, upon repeated exposure, to be associated with negative health outcomes ranging from internalizing disorders, including anxiety and depression, to cardiac and metabolic disease [4-9]. A better understanding of the dynamic day-to-day interplay between perceived stress and sleep duration could inform the mechanisms of their downstream impacts and the development of interventions to prevent or mitigate them [10-14]. Nevertheless, only a few studies have collected daily observations to evaluate within-person associations between stress responses and sleep duration, and their results are mixed: some studies report that heightened stress is followed by shorter sleep that night but not vice versa [15,16], others report that shorter sleep is followed by heightened stress the next day but not vice versa [17], and yet others report bidirectional relationships [18,19].

Inconsistent findings in stress-sleep associations might be at least partially explained by 2 interrelated methodological limitations, both of which are addressed by the individual-level modeling approach presented in this study. First, existing longitudinal studies have aggregated individual observations collected over short study periods of ≤14 days, thus limiting the estimation of stable associations that are robust to changing environmental demands (eg, first week of the semester vs final examinations period). Thanks to the adoption of digital phenotyping tools such as wearables and smartphones, research designs that sample individuals over much longer periods of time are increasingly feasible [20-22]. Sleep duration can now be passively tracked through continuous actigraphy sensing over months and even years, while perceived stress levels can be probed via brief daily smartphone-based surveys, with high compliance rates in student samples and small participant burden [23-25].

Moreover, the prevailing focus on group modeling and sample-level effects obscures the possibility that day-to-day associations between perceived stress and sleep duration may vary across individuals; for instance, it is possible that in certain individuals, getting less sleep than usual has no significant impact on stress levels the following day, but heightened stress during the day leads to shorter sleep duration later that night, while the opposite pattern might be true for others. Even when group-level models allow for individual-level estimates, such as in multilevel models with random effects, the degree to which the individual estimates are pulled toward the group (a *shrinkage* effect) is different for each individual based on the amount of data they provide, thus reducing the individual tailoring in a nonuniform manner [26].

Individual-level linear models (iLMs), which are fitted over a single individual’s intensive longitudinal observations, may offer a critical alternative to the estimation of stress-sleep associations. Compared to group-level approaches, iLMs provide estimates of phenotypes that are tailored to each person’s data and unbiased by the group [27-30]. They may also be more readily applicable in real-world contexts, where a clinician might use a precision health approach to evaluate and support each individual patient based on their personal data rather than on a hypothetical average patient [31-34]. Of note, individual-level approaches can also contribute to conclusions at the group or population level, such as by estimating the prevalence of each individually derived phenotype, rather than blurring across individuals, to arrive at a central tendency that might not accurately represent many of the included individuals.

### Objectives

In this study, we introduce a novel iLM approach that leverages daily observations collected over a full academic year for the assessment of day-to-day associations between actigraphy-derived sleep duration and self-reported stress levels in first-year college students. Our aims were 2-fold. First, we used a pilot data set to develop and validate a parsimonious iLM that estimates concurrent or lagged associations between 2 daily variables of interest while accounting for the weekly structure in student behavior. We then leveraged this iLM for the target examination of bidirectional day-to-day associations between perceived stress and objective sleep duration in an independent data set of 49 first-year college students. We expected a negative association for most participants—such that higher stress levels are associated with shorter sleep—but we also anticipated that some participants might show positive or null associations. We further hypothesized that the lead-lag relationship between perceived stress and sleep duration (ie, which of the 2 temporally precedes the other) would vary across individuals such that, for some, elevated stress would associate with shorter sleep that night; for others, shorter sleep would associate with elevated stress the next day; and for still others, both directions of association would be identified.

## Methods

### Participants

#### Pilot Group

A total of 6 undergraduate students returning for their sophomore year volunteered for a year-long study (all aged 19 years; n=3, 50% women and n=3, 50% men). All had participated in a previous pilot study in our laboratory during their first year of college and were known to be compliant. These 6 pilot participants provided data to develop the statistical models that were then applied to the new group of target participants described in the next subsection. The pilot participants enrolled for this study during the first 2 weeks of their fall semester. As in the case of the target participants, they were required to be taking full-time classes and own a smartphone compatible with the study smartphone app, Beiwe, which is part of the open-source Beiwe platform for digital phenotyping [35]. Available and missing data information at the participant level is provided in Figure S1 in Multimedia Appendix 1. Given our focus on individual-level models where each person serves as their own baseline, and there is no aggregation across participants, students were not excluded for current or past psychiatric disorders or medication use, and nor were they excluded if they began treatment or medication for mental health issues during the study.

#### Target Group

In total, 49 undergraduate students beginning their first year of college volunteered for a year-long study (aged 18-19 years; mean age 18.1, SD 0.24 y; n=25, 51% women and n=24, 49% men). We have previously reported data from these participants [24]. Participants living on campus were recruited via flyers posted on campus boards and distributed via email lists and were enrolled during the first 2 weeks concurrently with the pilot participants. Enrollment criteria were the same as for the pilot participants other than the fact that the target first-year participants were all new to the university. Initially, 68 participants enrolled, of whom 19 (28%) were excluded based on issues with data acquisition, including early withdrawal from the study (n=7, 37%), technical failure of the actigraphy data (n=1, 5%), poor-quality actigraphy data (n=2, 11%), and completion of <50% of the daily surveys (n=9, 47%). Of the 49 participants in the final sample, 2 (4%) identified as American Indian, 5 (10%) as Asian, 7 (14%) as Black, 31 (63%) as White, and 2 (4%) as mixed race. Furthermore, 12% (6/49) reported prior diagnosis of a psychiatric disorder (including anxiety, depression, and attention-deficit/hyperactivity disorder), and 8% (4/49) maintained active diagnoses. The 49 first-year students had not yet declared their area of study, but they reported their desired future occupation to be medicine (n=15, 31%), business or finance (n=7, 14%), academia or other research (n=6, 12%), engineering (n=5, 10%), policy or government (n=5, 10%), law (n=4, 8%), and other or undecided (n=7, 14%). Of the 49 participants, 46 (94%) were iPhone users, whereas 3 (6%) used Android mobile phones.

### Study Design

As previously reported in our study [24], this intensive longitudinal observational study collected passive and active data as participants engaged in their lives over a full academic year, extending a few days into the summer break. Both pilot and target participants completed smartphone-based daily surveys and a voice-recorded diary; wore an actigraphy wristband (GENEActiv Original; Activinsights Ltd) for continuous activity and sleep monitoring for the duration of the study; completed a battery of web-based questionnaires at the beginning, middle, and end of the study; and attended brief in-person check-ins every 3 to 4 weeks.

### Ethical Considerations

Informed consent and all study procedures and methods were approved by the institutional review board of Harvard University (IRB16-1230). All participants completed an in-person informed consent session where study procedures were explained, and any questions were clarified. Participants were informed that they could withdraw their study participation at any time. Participants were compensated US $1 per each daily survey they submitted, US $1 per day for continuously wearing the actigraphy wristband, and US $20 per hour for web-based surveys and attending in-person visits. Milestone bonus payments for completing half of the study (US $100) and the full study (US $300) were also provided to compensate participants for their continued compliance.

Study data collected across devices were stored and automatically backed up in a secure data warehouse configured to automatically import data from various collection streams. All data were kept as securely as possible and were only accessible to study staff. Participants’ data were labeled with a randomly generated participant ID. Personally identifying information, such as names and contact information, were kept separate from all other collected data in a locked file cabinet (in a locked office, behind an ID card–secured suite during off-hours) and in a password-protected database.

### Measures and Quality Control

#### Objective Sleep Duration

Daily sleep duration reflects the number of minutes between the estimated start and end of the day’s longest detected sleep episode. As redescribed from our original study [24], sleep duration was derived from the accelerometer data collected through the GENEActiv Original actigraphy wristband and analyzed via the deep phenotyping of sleep processing pipeline [36]. Participants wore the wristband on their nondominant wrist continuously, including during sleep and when bathing. Triaxial acceleration was collected with a sampling frequency of 30 Hz during the academic semesters and 10 Hz during the winter break (to extend battery life and memory while participants were away from campus). Participants were instructed to press the wristband’s button when they began trying to fall sleep at night and immediately after they awoke in the morning. Individuals exchanged their wristband for a fully charged one with reset memory at the in-person check-ins.

The deep phenotyping of sleep (DPSleep) processing pipeline was applied to the raw actigraphy data to detect the major sleep episode for each day [36]. The pipeline first converted the accelerometer data to minute-based activity estimates, removed the minutes when the individual was not wearing the device, and then estimated the major sleep episode based on a sliding window. Days where one of the boundaries of the sleep episode (ie, rises in relative activity both before and after a period of lower activity) could not be detected due to missing data were labeled as unusable. Two independent trained raters examined the automatically detected start and end times and the usability label of each sleep episode against the minute-based activity levels and the participant button presses when available. When necessary, they adjusted the automatic times and labels. Any disagreements between the 2 raters’ assessments were reviewed by the research team and resolved through discussion. A full description of the DPSleep processing pipelines applied to the actigraphy data, including quality control steps, can be found in the study by Rahimi-Eichi et al [36].

All data that passed quality control were included in analysis, including days with no detected sleep episode (ie, with no extended periods of lower relative activity).

#### Daily Telephone-Based Surveys

Smartphone surveys were administered via the Beiwe app [35]. Each night before bed, participants completed a 46-item self-report survey related to their daily lives. As described originally in our study [24], the questions assessed a range of behaviors and internal states over the past 24 hours, including sleep quality, stress levels and sources, positive and negative affect, general physical health, daily consumption habits, studying behaviors, and sociability and support [24]. This paper reports analyses using 2 survey questions selected a priori that probed daily subjective sleep quality and perceived stress. The sleep quality question asked “How did you sleep last night?” and was answered on a 5-point scale (1=terribly: little or no sleep, 2=not so well: got some sleep but not enough, 3=sufficient: got enough sleep to function, 4=good: got a solid night’s sleep and felt well rested, and 5=exceptional: one of my best nights of sleep). The perceived stress question asked “How much did you feel stressed over the past 24 hours?” and was also answered on a 5-point scale (1=very slightly or not at all, 2=a little, 3=moderately, 4=quite a bit, and 5=extremely).

Surveys submitted between 5 PM (local time) on the day the survey opened and 6 AM the following day were considered to be on time. Surveys submitted after 6 AM the day after the survey was prompted were marked as missing. A participant was included in analysis if they were compliant with at least 100 daily surveys across the data collection period, and only on-time surveys from these participants were included.

### Analytical Approach

#### Development of the iLM

An iLM was developed on the intensive day-level longitudinal data from the 6 pilot participants to test person-specific day-to-day behavioral associations. The iLM framework allows for individually tailored estimates by treating each day as the unit of observation and the individual as the *population*, as opposed to each individual as the unit of observation used to estimate the general population. An individual’s observed time series data can be understood as just one realization of a stochastic process whose data-generating process we are trying to model and understand [37], in this case through linear models. The intercept and slope estimates are unique to the individual, and we interpret them as a proxy for the individual’s phenotype. Of note, in this framework, each individual model can be interpreted as an independent test of the hypothesized association (eg, is shorter sleep than usual associated with heightened perceived stress the following day?).

The model was structured to be parsimonious while accounting for the nonindependence of the daily measures and the temporal structure imposed by the academic schedule. The day of the week was included as a categorical covariate to account for weekend versus weekday effects and other weekly structures imposed by the college schedule (eg, classes that meet on Monday-Wednesday-Friday vs those that meet on Tuesday-Thursday). In addition, because behavioral patterns during the semester vary substantially from those during the 5-week winter break (when students do not have classes and typically are away from campus), we decided a priori to only include in the model compliant data collected during the school semesters.

The final iLM took the following general form:

y_t_ = β0 + β1x_t_ or _t−1_ + β2DayOfWeek_t_ + ε_t_

A daily observation on day*_t_* starts with the nighttime sleep episode and ends with the submission of the daily survey submitted in the evening before the next sleep episode. In the aforementioned formula, *y_t_* is a single participant’s outcome variable (eg, sleep duration in min) at daily time point *t*, *β_0_* is this participant’s individual intercept, *x* is the predictor variable (eg, sleep quality or stress measured on a 1-5 scale) at daily time point *t* or *t−1* (depending on the lag of the tested association), *β_1_* is the participant’s individual slope for *x*, *DayOfWeek_t_* is the day of the week in which the outcome observation was acquired (modeled as a categorical variable ordered from Saturday to Friday), and *ε_t_* is a normally distributed random error term.

All modeling was carried out in a Bayesian inference framework, which treats unknown parameters (eg, a slope) as random variables with a probability distribution rather than a discrete value; this distribution is updated based on the observed data (resulting in a *posterior distribution*) and serves as a measure of uncertainty around the parameter [38-40]. The Bayesian framework was favored for these analyses due to its flexibility in computing models with varied specifications (including complex random effect specifications in the multilevel models we fitted as part of our model validation process), robustness to sample data characteristics (eg, dispersion), and intuitive interpretation of the posterior distribution and 95% uncertainty interval (UI; ie, conditional on the data and the model, the probability that the parameter is contained in the interval is 0.95) [39,40]. For comparison, parallel iLMs fitted in a frequentist inference framework in the pilot validation stage yielded nearly identical point estimates to their Bayesian counterparts (refer to Figure S2 in Multimedia Appendix 1), suggesting that our model specification is robust across both statistical inference frameworks. All models were estimated in R (version 4.3.1; R Foundation for Statistical Computing) [41]. Bayesian models were estimated using the Stan modeling language [42] and the packages *rstanarm* (version 2.21.4 [43]), *tidybayes* (version 3.0.1 [44]), and *bayestestR* (version 0.13.1 [45]). Frequentist iLMs were estimated using the *stats* package included in base R [41].

Bayesian models were fit with default weakly informative priors specifying a gaussian distribution (mean 0, SD 2.5) to represent our diffuse prior knowledge. We estimated parameters using a Markov chain Monte Carlo (MCMC) approach. For each parameter, we sampled from 4 stationary Markov chains, each comprising 5000 sampling iterations, including a burn-in period of 2500 iterations that were discarded (for a total of 10,000 post–warm-up draws). Convergence of the 4 chains to a single stationary distribution was assessed quantitatively via the R-hat convergence diagnostic [46] (adequate convergence defined as R-hat <1.1) and qualitatively by visual inspection of trace plots showing the estimated parameter as a function of each chain’s iteration number (adequate convergence defined as the chains overlapping with each other throughout and a lack of structured patterns in each chain). Each model’s effective sample size (ESS) metric is reported. Each estimate in the MCMC process is serially correlated with the previous estimates: the higher the correlation, the more samples are needed to get to a stationary distribution. In the presence of nearly no autocorrelation, the ESS will be equal to the number of posterior draws requested (in this case, 10,000). Generally, the ESS should be at least 1000 to obtain stable estimates [38,47].

Adequacy of the model specification was assessed via 2 methods. First, posterior predictive checks entailed a visual comparison of the distribution of the observed outcome variable to the distribution of 100 simulated outcome data sets generated by applying 100 draws from the model parameters’ posterior distribution to our observed data set. Similarity in the distributions of the observed and model-generated outcomes suggests that the model specification captured the data well. Second, we inspected the model residuals against the model’s predicted values to confirm homoscedasticity and against themselves to rule out problematic autocorrelation due to temporal dependencies in longitudinal data.

Point estimates of intercepts and slopes were computed as the median value of their respective posterior distributions. Furthermore, 95% UIs were computed as the 2.5% and 97.5% quantiles of the posterior distribution. To provide intuitive parallels to a frequentist inference framework, we interpreted a predictor slope as statistically significant if its 95% UI did not contain 0, or, put differently, if the proportion of the posterior distribution falling in the direction (positive or negative) of the point estimate (also known as *probability of direction* [pd]) was higher than 0.975 (which approximates a frequentist 1-tailed *P* value of <.025) [48]. Although we focus on 95% as the cutoff for the UI given the widespread use of this number in the literature, it should be noted that this threshold has been criticized as arbitrary [39]. To go beyond testing whether the slope is different from exactly 0, we also report the percentage of the 95% UI that falls within a *region of practical equivalence* (ROPE), defined as parameter values that are sufficiently close to 0 to be considered equivalent to the null for practical purposes [49] and mathematically defined as a standardized effect size of <0.1 (ie, half of a *small effect* as defined by Cohen [50]). Given our individualized approach, ROPEs were estimated separately for each participant based on their observed data.

#### Validation of the iLM in the Pilot Data

Data from 6 pilot participants were used to test the properties of the iLM and explore associations among variables of interest to validate the approach. A first sanity check was to assess whether the iLM captured the expected association between a sleep event’s objective duration (estimated from an actigraphy wristband) and the subjective rating (reported at the end of each day as part of a smartphone-based survey) for the same (*concurrent*) sleep event. Participants were expected to rate episodes of shortened sleep as worse quality compared to nights of longer sleep. This contemporaneous model, known as a static model in the time series literature [37], was specified as follows:

ObjectiveSleepDuration_t_ = β_0_ + β_1_SubjectiveSleepRating_t_ + β_2_DayOfWeek_t_ + ε_t_

A second, exploratory lagged model was fit testing associations between sleep duration on day_t_ and sleep quality reported the day before, on day*_t−1_*. We expected that this lagged association would be weaker than the concurrent association outlined previously (given that the variables tested are no longer referring to the same sleep event), and importantly, that the association would be in the opposite direction such that poor-quality sleep on one night is associated with longer sleep duration the next night, signaling a compensatory sleep rebound effect. To test this, a model similar to the aforementioned one was fit, with the difference that the predictor was lagged by 1 day. This lagged model, known as a finite distributed lag model in time series analysis [37], was specified as follows:

ObjectiveSleepDuration_t_ = β_0_ + β_1_SubjectiveSleepRating_t−1_ + β_2_DayOfWeek_t_ + ε_t_

Both models were fit with a gaussian distribution to reflect the observed normal distribution of sleep duration in our sample.

We conducted model checks to evaluate the performance of the iLM framework. Posterior predictive checks (described earlier in this subsection) evaluated that a gaussian model specification captured the distribution of the data well. In addition, given the longitudinal design, we examined whether the model residuals lacked meaningful autocorrelation, which would suggest that our day-of-the-week covariate sufficiently captured the weekly structure of sleep duration. Finally, we compared the estimates obtained through the iLMs to the estimates obtained when the same data for the 6 pilot participants were fit within a single, group-based multilevel linear model (MLM), specifying fixed effects for intercepts and slopes and additional participant-level random intercepts and random slopes for the main predictor of interest (in this case, sleep quality). This comparison allowed us to assess our expectation that MLMs would provide individual estimates roughly comparable to those of the iLM, but with the critical difference that MLMs would systematically attenuate these individual estimates, especially for individuals who deviate from the predominant association phenotype or when there is large heterogeneity in these phenotypes across individuals.

#### Application of the iLM to the Novel Target Participants

After developing the iLM and validating it extensively over the pilot data set, the modeling framework was then carried forward and applied to the independent target data set of first-year college students. All models were fit with a gaussian distribution. We first applied the same sanity check models as in the pilot data set. Subsequently, we used the iLM method to test a priori target hypotheses regarding the association between perceived daily stress and objective sleep duration.

Two target models were fit for each individual to examine bidirectional associations between sleep duration and perceived stress. A daily observation on day*_t_* consists of last night’s sleep duration (*ObjectiveSleepDuration_t_*, recorded passively via the actigraphy wristband) and the subjective rating of the present day’s overall stress levels (*SubjectiveStressRating_t,_* reported by the participant in the evening). First, to test whether stress level during the day is related to sleep duration later that night (ie, a *stress-then-sleep* association), the model used the stress rating the day before sleep (day*_t−1_*) as the predictor of subsequent sleep duration (day*_t_*). Thus, the formula for this model was specified as follows:

ObjectiveSleepDuration_t_ = β_0_ + β_1_SubjectiveStressRating_t−1_ + β_2_DayOfWeek_t_ + ε_t_

Next, to test whether sleep duration is related to stress levels the day after (ie, a *sleep-then-stress* association), the model used stress rating the day after sleep (day*_t_*) as predictor of the previous sleep duration (day*_t_*, sleep duration last night):

ObjectiveSleepDuration_t_ = β_0_ + β_1_SubjectiveStressRating_t_ + β_2_DayOfWeek_t_ + ε_t_

In this model, stress rating (predictor) is back-predicting sleep duration (outcome), which effectively tests the question “Is stress today associated with sleep last night?” As the objective sleep duration measure occurs temporally before the daily stress rating is submitted, results are interpreted as sleep duration being associated with increased or decreased stress the next day. Implementing the model in this way (rather than using sleep duration as the predictor and stress rating as the outcome) presented the advantage that the slope estimates across the 2 models are on equivalent units, namely, the change in the number of minutes in sleep duration per unit of change in stress rating. This allows us to directly compare the quantitative outputs for the models testing the questions “Is stress associated with subsequent sleep?” and “Is sleep associated with subsequent stress?”

As with the pilot data set, we conducted model checks to further evaluate the specification and performance of the iLM framework in our target data set, including posterior predictive checks; inspection of model residuals; and a comparison of the estimates obtained through the iLMs and the estimates obtained when the same data for the target sample were fit in a single, group-level MLM with random intercepts and random slopes per participant on the stress predictor. Additional inspection of model results against each participant’s raw time series data was conducted to complement our interpretation.

## Results

### Participant-Level Descriptive Statistics

Table 1 presents participant-level available data and summary statistics for sleep and stress variables used in analysis. The pilot participants provided a median of 178 (range 119-212 out of a total possible of 223) usable observations for modeling, that is, day-level observations collected during the school semesters with usable actigraphy and survey data. The target participants provided a median of 178 (range 84-214) usable observations. Participants’ total number of usable observations was not correlated with their mean sleep duration (*r*=0.09; *P*=.53) or mean stress levels (*r*=−0.11; *P*=.42). In addition, participants’ mean sleep duration did not differ on days with or without available survey data (paired 2-tailed *t* test, *P*=.59), and participants’ mean stress levels did not differ on days with or without available actigraphy data (paired 2-tailed *t* test, *P*=.70). These observations suggest that participants’ overall sleep and stress metrics did not introduce systematic missingness in the data (more details on participant-level missing data are presented in Figure S1 in Multimedia Appendix 1).

**Table 1:** Participant-level descriptive statistics of data used in analyses.

Participant	Daily observations	Sleep duration (min), mean (SD)	Sleep quality (1-5 Likert scale), mean (SD)	Stress (1-5 Likert scale), mean (SD)
P1^a^	119	493.22 (101.89)	3.59 (0.64)	1.36 (0.74)
P2	212	412.08 (84.57)	2.50 (0.68)	2.95 (0.82)
P3	156	404.03 (68.81)	2.48 (0.55)	2.89 (0.93)
P4	200	425.20 (82.42)	2.64 (0.69)	3.15 (0.42)
P5	200	387.76 (58.32)	3.14 (0.74)	2.23 (1.14)
P6	147	379.29 (139.37)	2.65 (1.12)	3.85 (1.14)
T1^b^	147	464.92 (96.66)	3.81 (0.94)	2.76 (1.06)
T2	212	460.47 (82.68)	3.08 (0.62)	3.16 (0.83)
T3	139	415.09 (114.00)	2.61 (0.71)	3.59 (0.82)
T4	103	349.96 (89.02)	2.81 (0.83)	1.69 (1.09)
T5	189	446.40 (60.47)	3.33 (0.62)	2.51 (0.83)
T6	199	465.13 (76.76)	3.07 (0.63)	2.68 (0.77)
T7	131	389.17 (56.96)	3.06 (0.91)	1.95 (1.19)
T8	173	402.33 (108.91)	2.87 (0.49)	2.69 (0.63)
T9	102	448.53 (144.97)	2.95 (0.79)	1.98 (1.01)
T10	208	432.88 (114.48)	2.71 (0.73)	3.07 (1.11)
T11	173	373.92 (94.30)	2.62 (0.73)	2.77 (0.78)
T12	87	475.03 (75.76)	2.83 (1.00)	3.57 (1.14)
T13	214	441.09 (73.22)	2.49 (0.56)	3.25 (0.70)
T14	84	454.23 (100.68)	3.40 (0.62)	2.52 (0.81)
T15	98	412.93 (64.32)	2.57 (0.66)	3.41 (0.87)
T16	152	414.31 (127.89)	2.70 (0.66)	3.08 (0.83)
T17	191	440.73 (74.20)	3.06 (0.50)	1.37 (0.68)
T18	92	385.57 (70.90)	3.09 (0.51)	2.87 (0.63)
T19	199	464.09 (74.95)	2.87 (0.44)	2.94 (0.59)
T20	192	497.65 (77.78)	3.69 (0.58)	2.30 (0.95)
T21	199	390.89 (82.58)	3.36 (0.76)	2.85 (1.05)
T22	126	464.25 (84.70)	3.33 (0.90)	2.53 (1.04)
T23	84	418.65 (73.60)	2.87 (0.62)	3.39 (0.79)
T24	148	482.32 (81.06)	3.19 (0.87)	2.01 (0.77)
T25	191	406.52 (95.58)	2.70 (0.78)	3.47 (1.00)
T26	205	446.68 (64.03)	3.01 (0.10)	2.94 (1.14)
T27	177	443.10 (114.21)	3.16 (0.82)	3.28 (0.76)
T28	209	409.12 (65.85)	2.92 (0.59)	2.67 (0.91)
T29	197	429.07 (74.85)	3.00 (0.47)	1.68 (0.88)
T30	195	396.16 (62.76)	2.67 (0.59)	2.85 (1.05)
T31	205	388.53 (94.91)	2.82 (0.83)	2.52 (0.71)
T32	117	397.49 (118.98)	2.61 (0.86)	3.73 (1.10)
T33	114	383.05 (135.02)	2.65 (0.89)	2.09 (1.27)
T34	206	436.76 (80.84)	2.88 (0.73)	2.43 (0.89)
T35	207	461.99 (71.50)	3.08 (0.78)	3.16 (1.23)
T36	188	470.90 (86.02)	3.36 (0.80)	2.70 (1.10)
T37	180	423.49 (78.52)	3.41 (0.63)	2.21 (0.85)
T38	139	409.22 (132.79)	2.56 (0.85)	3.39 (0.82)
T39	114	414.25 (99.79)	2.74 (0.67)	2.25 (1.07)
T40	102	483.04 (53.83)	3.37 (0.53)	2.84 (1.24)
T41	136	429.99 (91.77)	2.34 (0.67)	3.54 (0.74)
T42	193	416.79 (95.16)	3.26 (0.80)	1.21 (0.46)
T43	166	464.57 (116.07)	3.04 (0.35)	2.23 (0.82)
T44	179	456.01 (67.63)	3.34 (0.74)	1.96 (1.03)
T45	197	474.08 (64.90)	2.99 (0.60)	1.18 (0.54)
T46	203	478.48 (70.13)	1.99 (0.58)	2.35 (1.01)
T47	178	382.32 (78.80)	3.54 (0.72)	1.79 (0.95)
T48	189	406.65 (103.77)	2.81 (1.00)	2.17 (1.36)
T49	171	444.64 (87.14)	2.91 (0.86)	1.90 (1.11)

^a^IDs starting with “P” indicate pilot sample participants.

^b^IDs starting with “T” indicate target sample participants.

### Sleep and Stress Fluctuate in Relation to the Academic Calendar in the Pilot Participants

**Figure 1:**
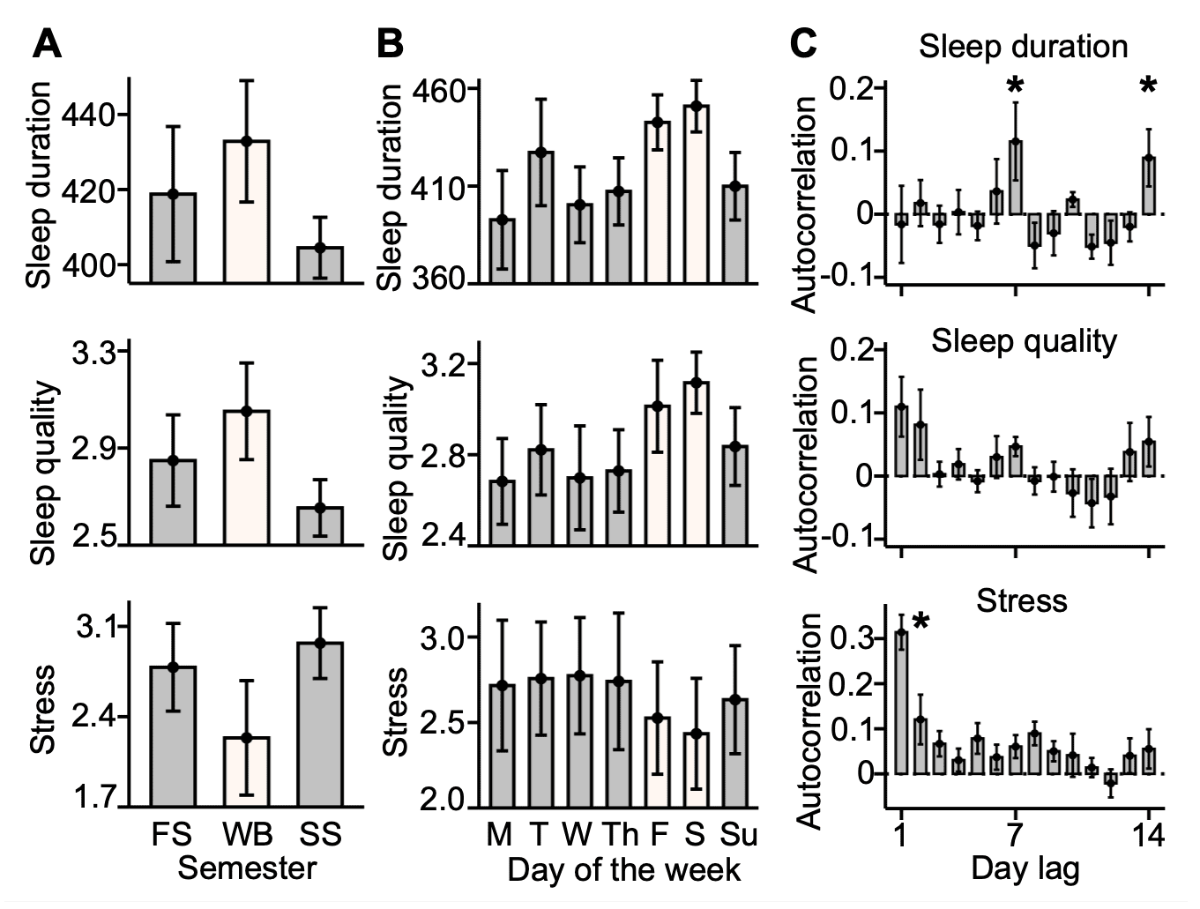
Group sleep and stress metrics fluctuate with the academic calendar. (A) The pilot participants’ group (between-person) means of sleep duration (in min), sleep quality (1-5 Likert scale), and perceived stress (1-5 Likert scale), aggregated by school semester (fall and spring) and winter break and (B) by the day of the week. (C) Between-person means of autocorrelation estimates over a 14-day window. Error bars show SE of the mean. *Autocorrelations were strongest at a 7-day lag (and again at a 14-day lag) for sleep duration and at 1-day and 2-day lags for perceived stress. F: Friday; FS: fall semester; M: Monday; S: Saturday; SS: spring semester; Su: Sunday; T: Tuesday; Th: Thursday; W: Wednesday; WB: winter break.

There was a pattern of enhanced sleep and lower stress when students were released from the structured academic demands of the in-person school semesters. Participants were enrolled for a full academic year, including a fall semester and a spring semester (each lasting roughly 16 weeks), as well as a 5-week class-free winter break bridging the 2 semesters. During the winter break and weekends, pilot participants had longer objective (actigraphy-derived) sleep duration, better subjective sleep quality, and felt less stress compared to school semesters and weekdays (Figures 1A and 1B). The temporal structure of sleep and stress variables was further evidenced by their autocorrelation estimates. Autocorrelations were generally small (|r|<0.2; Figure 1C) but were strongest (highlighted with asterisks) at a 7-day lag (and again at a 14-day lag) for sleep duration, consistent with a weekly sleep schedule. By contrast, autocorrelations for stress were strongest at 1- and 2-day lags, suggesting that experiences of stress might come in chains of >1 day.

The school break and week-related changes observed in the data informed the design of the iLM seeking to capture stable person-level associations. Given that the winter break presents different environmental demands from the school semester, we decided a priori to exclude from the model the observations collected during this period. In addition, to account for weekly patterns in sleep behavior (outcome variable) within the school semesters, we added the day of the week as a covariate in the model.

### iLMs Capture Day-to-Day Associations in the Pilot Participants

**Figure 2:**
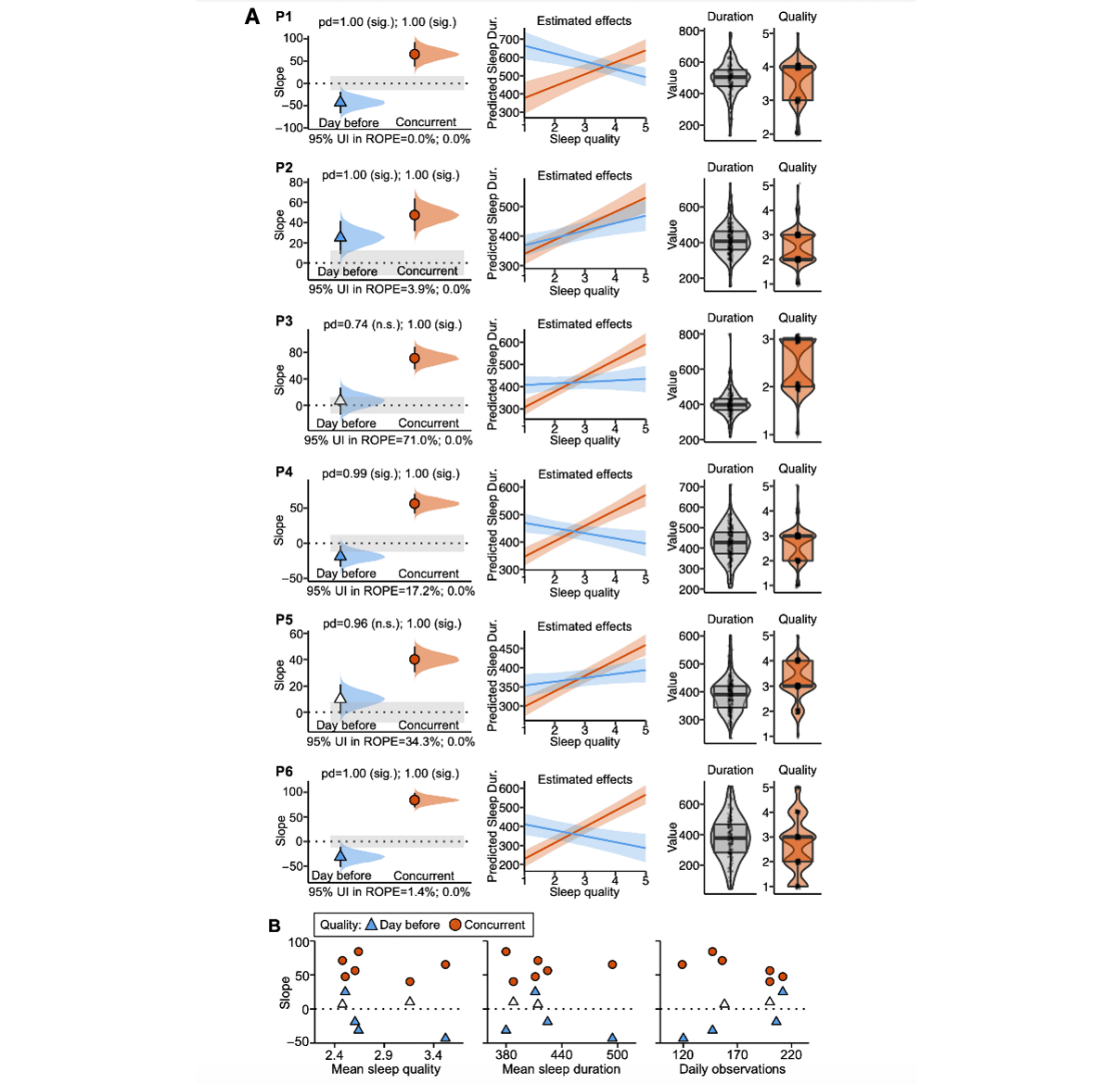
Within-individual linear models capture real-world associations between objective sleep duration and subjective sleep quality. (A) Each row (from participant 1 [P1] to participant 6 [P6]) shows model results for an individual pilot participant. Individual-level models testing sleep duration associated with sleep quality the day before are displayed in blue; models testing sleep duration associated with concurrent sleep quality are displayed in orange. Column 1 shows the models’ estimated slopes (computed as the median of the posterior distribution) and uncertainty metrics. Symbol shading signifies statistically significant slopes. Error bars show 95% uncertainty intervals (UIs). Shaded density plots show the full posterior distributions of the slopes; gray shaded areas show the regions of practical equivalence (ROPEs). Column 2 shows the models’ predicted sleep duration as a function of sleep quality; shading around the lines indicates 95% UIs. Column 3 shows the participant-level distributions of sleep duration (in min) and sleep quality (1-5 Likert scale) across all daily observations used in analysis. (B) Slope estimates from column 1 in subpart A are plotted against participant-level estimates across the study period. Dur.: duration; n.s.: not statistically significant; pd: probability of direction; sig.: statistically significant.

To test the viability of the iLM approach, a proof-of-concept model examined the association between objective sleep duration and subjective sleep quality in the pilot participants (Table 2 and Figure 2). This model allowed for a test of construct validity, given that the tested association was intuitive and expected. The model tested the association between sleep duration and sleep quality for the same sleep episode (ie, a *concurrent* association). All pilot participants showed the expected positive association: when participants slept for a shorter period than usual, they also subjectively rated these same sleep events as worse quality (shown in orange in Figure 2A). The effect size was large for all individuals, and the 95% UI lay outside of the ROPE for all models. These results provided preliminary evidence that the iLM is a valid framework to reliably detect relations between psychological variables (in this case, subjective sleep quality rating) and objective behaviors (sleep duration) at the individual participant level.

A second, exploratory iLM tested the association between sleep duration (on day_t_) and sleep quality the day before (on day_t−1_). We expected this model to show significant but weaker effects compared to the first model, given that the variables were referring to lagged sleep events. Moreover, we expected negative associations such that worse sleep quality on one night would associate with longer sleep duration the next night, that is, a compensatory sleep rebound effect. Half of the participants (3/6, 50%; P1, P4, and P6) showed this expected pattern of association (pd>0.975; shown in blue in Figure 2). Of the 6 participants, 1 (17%; P2) showed a positive association such that worse subjective sleep quality experienced on the preceding night was associated with shorter sleep durations on the subsequent night. This participant might experience chains of poor sleep over multiple days (eg, reduced sleep days ahead of a deadline to accommodate increased workload), rather than a sleep rebound effect immediately the following night. In addition, of the 6 participants, 2 (33%; P3 and P5) showed a positive slope estimate but without reaching statistical significance (pd<0.975). We did not observe structured patterns of association between individual slope estimates and individuals’ mean sleep quality, mean sleep duration, or total number of daily observations (Figure 2B). This suggests that the estimated slopes were not systematically influenced by person-level characteristics of the psychological and behavioral phenomena of interest.

**Table 2:** Pilot sample results of individual-level linear models assessing sleep duration associated with concurrent sleep quality and assessing sleep duration associated with sleep quality the day before (all models included the day of the week as a covariate, but the table only shows model parameters for the main predictor, sleep quality).

Model and pilot participant			Slope (median of the posterior distribution)		95% UI^a^		pd^b^		ROPE^c^ (+/−)		% UI in ROPE		R-hat		ESS^d^
**Sleep duration regressed on concurrent sleep quality**															
	P1	65.31^e^		37.73 to 92.42		1.00		15.84		0.00		1.00		7291	
	P2	47.48^e^		31.42 to 63.87		1.00		12.35		0.00		1.00		8177	
	P3	71.10^e^		54.30 to 88.46		1.00		12.50		0.00		1.00		7484	
	P4	56.32^e^		42.31 to 70.24		1.00		12.00		0.00		1.00		7649	
	P5	40.22^e^		30.65 to 49.95		1.00		7.89		0.00		1.00		7030	
	P6	84.28^e^		69.58 to 99.15		1.00		12.50		0.00		1.00		6578	
**Sleep duration regressed on sleep quality the day before**															
	P1	−43.28^e^		−67.61 to −18.99		1.00		15.84		0.00		1.00		7726	
	P2	25.05^e^		8.84 to 41.57		1.00		12.35		3.93		1.00		8244	
	P3	6.80		−14.01 to 27.05		0.74		12.50		71.04		1.00		7478	
	P4	−18.92^e^		−33.78 to −3.38		0.99		12.00		17.20		1.00		7823	
	P5	10.07		−1.05 to 21.18		0.96		7.89		34.29		1.00		8030	
	P6	−31.39^e^		−51.90 to −10.44		1.00		12.50		1.36		1.00		6911	

^a^UI: uncertainty interval.

^b^pd: probability of direction.

^c^ROPE: region of practical equivalence.

^d^ESS: effective sample size.

^e^Statistically significant result.

### Model Diagnostics Confirm Convergence and Adequate Specification

**Figure 3:**
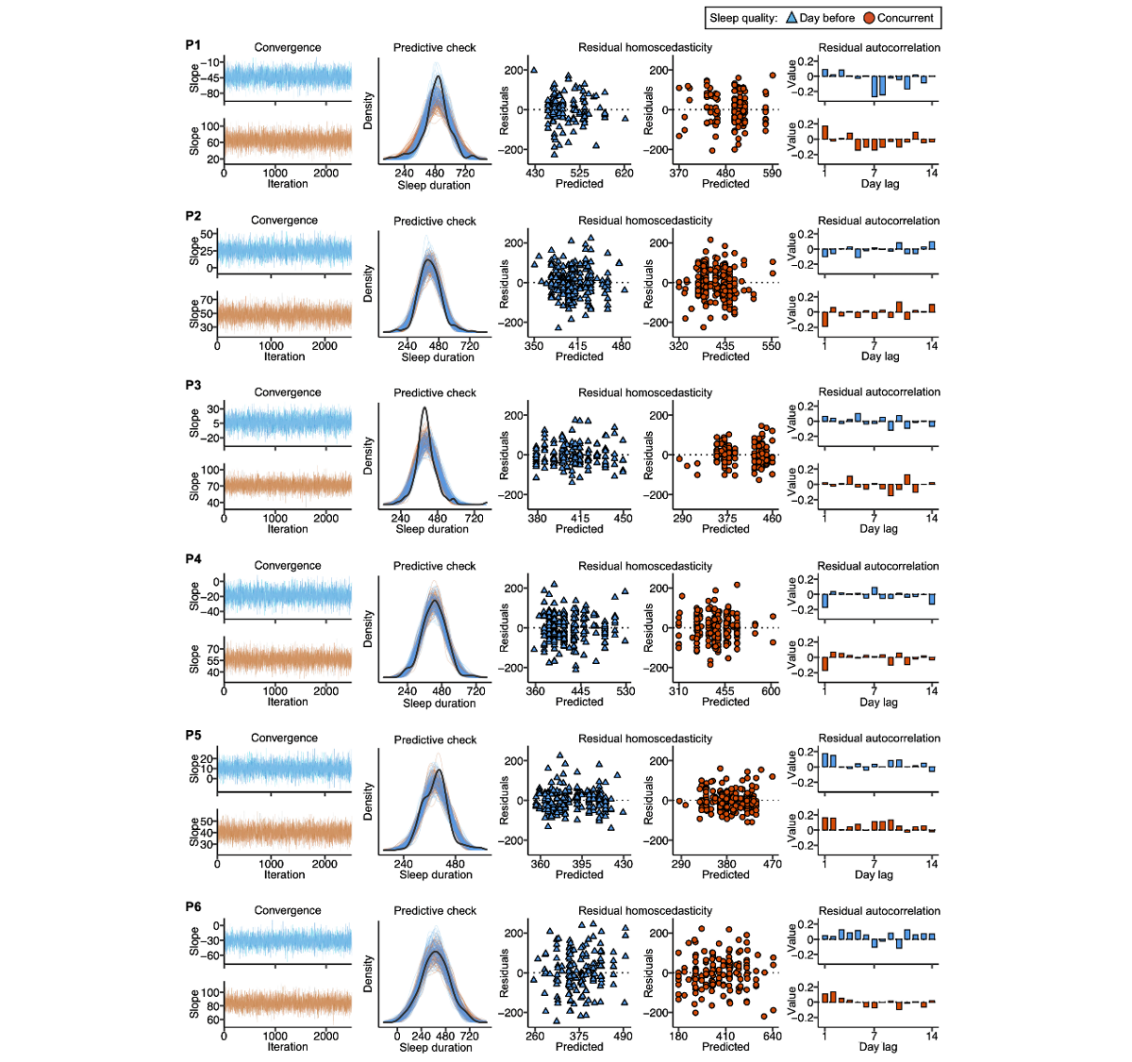
Within-individual model diagnostics suggest adequate convergence and specification. Each row (from participant 1 [P1] to participant 6 [P6]) shows model diagnostics for an individual pilot participant. Diagnostics for the models assessing the association between sleep duration and sleep quality the day before are displayed in blue; models assessing the association between sleep duration and concurrent sleep quality are displayed in orange. Column 1’s trace plots display time series of each Markov chain’s estimated slope (y-axis) as a function of postwarmup iterations (x-axis). Column 2 shows posterior predictive checks; black density lines show the observed distributions of the outcome (sleep duration), and the thin colored density lines show 100 replicated outcome distributions generated based on random samples from the models’ parameters’ posterior distributions. Columns 3 and 4 show the models’ predicted values (x-axis) against the models’ residuals (y-axis). Column 5 shows autocorrelation plots of model residuals.

Both visual and quantitative MCMC diagnostic checks revealed that all iLMs converged successfully (Figure 3). Trace plots (Figure 3, column 1) revealed no structured pattern in the estimated slopes across sampling iterations. R-hat values were <1.1, and ESSs were >1000 for the estimated slopes of all models (Table 2). Posterior predictive checks comparing the observed distribution of the outcome variable (sleep duration) to 100 randomly sampled simulated data sets from the posterior predictive distribution confirmed that a gaussian model specification captured the observed data well (Figure 3, column 2). Inspection of the model residuals against the model’s predicted values confirmed homoscedasticity, with no structured pattern in the plots (Figure 3, columns 3 and 4). Finally, residual autocorrelation was generally low (|*r*|<0.2) for all models (Figure 3, column 5), suggesting that the model specification was able to account for the temporal structure in the data.

### iLMs Yield Similar, but Not Identical, Estimates to a Group-Based Multilevel Model

**Figure 4:**
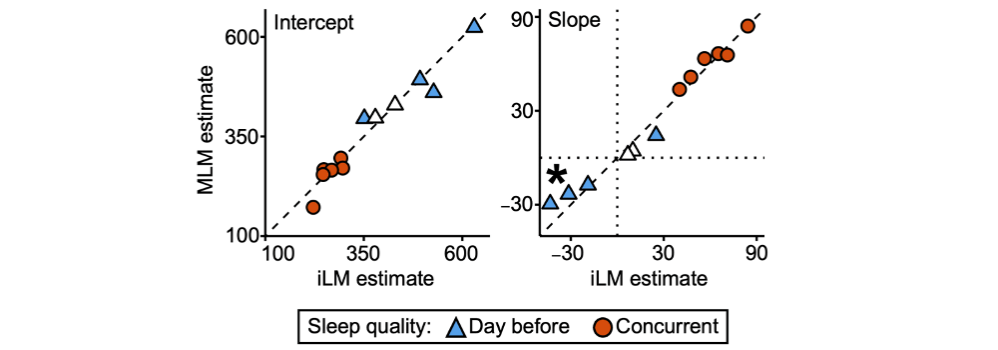
Within-individual linear models of the association between sleep duration and sleep quality yield similar, but not identical, estimates to a group-based linear model. Estimates of the intercepts and slopes from the individual-level linear models (iLMs; x-axis) for each participant are compared to the random effects estimated from a group-based multilevel linear model (MLM; y-axis). Triangles show estimates for the models examining the association between sleep duration and sleep quality the day before; circles show estimates for the models examining the association between sleep duration and concurrent sleep quality. Symbol shading signifies statistically significant slopes in the iLMs. Dashed diagonal lines represent identical estimates between the individual- and group-based approaches. *Attenuation of individual estimates in the MLM compared to the iLMs.

The slopes estimated from the iLM are similar to those obtained through a MLM testing the same associations between concurrent objective sleep duration and subjective sleep quality (Figure 4). This suggests that the individually tailored slopes obtained from our iLM are comparable to more traditional group-based approaches. Of note, although the estimates are similar, they are not identical.

Comparison of the 2 models suggested attenuation of the individual-level estimates in the group models (refer to the part of Figure 4 highlighted with an asterisk). Even with random effects by participant, in the MLM, these estimates are, by design, biased by the group and may underestimate individual-level effects, especially for uncommon phenotypes [20]. As will be revealed later in the results from the larger sample of target participants, group-based estimation can even result in the reversal of the sign of the association for some individuals.

### The iLM Is a Valid Framework to Identify Individual-Level Associations

Proof-of-concept analyses of the pilot data demonstrated that the iLM successfully captures expected associations between objective behavior and psychological variables, has an adequate model specification, and captures individual-level estimates unbiased by the group. Moreover, point estimates obtained with our Bayesian iLMs are nearly identical to those obtained through iLMs fitted with a frequentist inference framework (refer to Figure S2 in Multimedia Appendix 1), suggesting that our individual-level modeling approach is robust across both major statistical inference frameworks. In sum, the iLM is a valid framework to identify individual-level associations, which justified carrying over the model to test target hypotheses regarding the association between perceived stress and objective sleep duration in the independent target data set of first-year students (n=49).

### Sleep and Stress Fluctuate With the Academic Calendar in the Target Participants

As with the pilot participants, during winter break and weekends, participants had longer objective sleep duration, better subjective sleep quality, and felt less stressed compared to the fall and spring semesters (columns 1 and 2 in Figure S3 in Multimedia Appendix 1). We replicated the autocorrelation pattern seen in the pilot data set: sleep duration showed the greatest autocorrelation at a 7-day lag (and then again at a 14-day lag), indicating that sleep patterns are tied to a weekly schedule, while stress showed the greatest autocorrelation at 1-day and 2-day lags, indicating that experiences of stress might come in chains of a few days (column 3 in Figure S3 in Multimedia Appendix 1). These school break– and week-related dynamics confirm that our target data set captured real-life dynamics associated with college life and reinforce our individual-level modeling decisions regarding the exclusion of winter break data and the addition of the day of the week as a covariate.

### Shorter Objective Sleep Duration Is Associated With Worse Subjective Sleep Quality in the Target Participants

**Figure 5:**
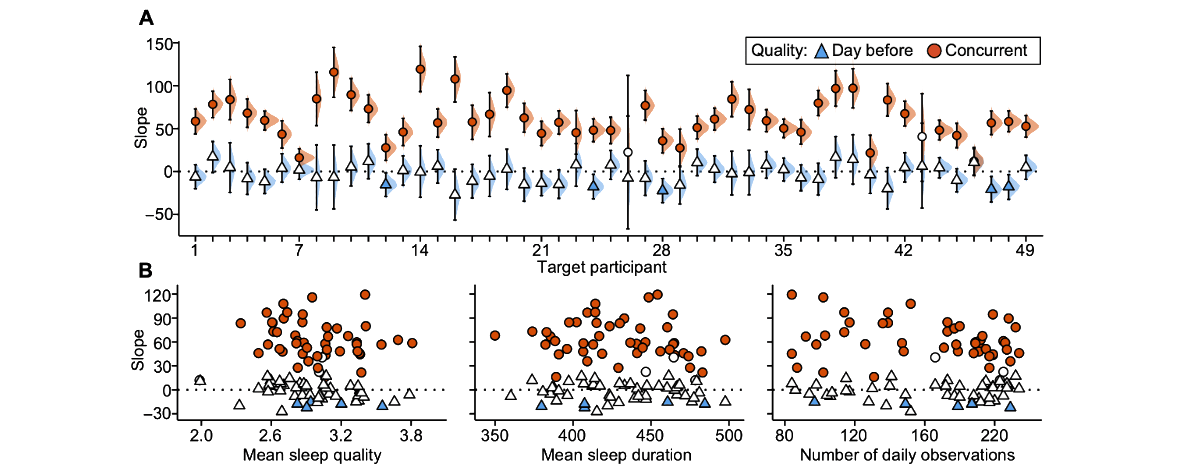
Longer objective sleep duration associates with higher subjective sleep quality in most individuals. (A) Estimated slopes (corresponding to the median of the posterior distribution) from the individual-level models that assess sleep duration associated with sleep quality the day before (triangles) and with concurrent sleep quality (circles) are plotted along the y-axis ordered by participant (x-axis). Statistically significant slope estimates are shaded blue (day before) or orange (concurrent). Error bars show 95% uncertainty intervals, and shaded density plots show the full posterior distributions of the slopes. (B) Slope estimates from subpart A are plotted against participant-level estimates across the study period.

All participants showed a positive slope estimate for the association between concurrent sleep duration and sleep quality, and this effect was statistically significant in 46 (94%) of the 49 target participants (pd>0.975; Table 3 and Figure 5A, in orange). In other words, when participants slept for a shorter period than usual, they also rated these same sleep events as worse quality, demonstrating that the iLM can capture expected real-world relations between behavioral and psychological phenomena. Among participants who showed a significant association (46/49, 94%), a 1-point increase in a 5-point sleep quality scale was associated with a sleep episode that was also longer by a median 59 (range 16-119) minutes, a substantial increase in sleep duration for most participants considering that the average sleep duration in the sample was 432 minutes (7.2 h; SD 34 min).

For the exploratory lagged model, the expected negative association between sleep duration and sleep quality the day before reached statistical significance in only 5 (10%) of the 49 participants (pd>0.975; Table 4 and Figure 5A, in blue). In other words, for only 10% of participants, sleep rated as worse quality was consistently followed by longer sleep the following night, suggesting that sleep rebound effects are perhaps less common or reliable than anticipated. Among these participants, a 1-point decrease in a 5-point sleep quality scale was associated with a subsequent sleep episode that was longer by a median 18 (range 15-22) minutes across participants, a much smaller effect compared to the concurrent model. We did not observe structured patterns of association between individual slope estimates and individuals’ mean sleep quality, mean sleep duration, or total number of daily observations, suggesting that the model results were not systematically influenced by person-level aggregates of the variables that went into the model (Figure 5B).

**Table 3:** Target sample results of individual-level linear models assessing sleep duration associated with concurrent sleep quality (all individual-level models included the day of the week as a covariate, but the table only shows model parameters for the main predictor, sleep quality).

Target participant	Slope (median of the posterior distribution)	95% UI^a^	pd^b^	ROPE^c^ (+/−)	% UI in ROPE	R-hat	ESS^d^
T1	58.57^e^	43.90 to 72.99	1.00	10.30	0.00	1.00	7523
T2	78.35^e^	62.80 to 93.58	1.00	13.36	0.00	1.00	8984
T3	83.88^e^	60.42 to 107.40	1.00	16.12	0.00	1.00	7211
T4	68.08^e^	51.48 to 84.48	1.00	10.74	0.00	1.00	7738
T5	59.57^e^	48.38 to 70.46	1.00	9.77	0.00	1.00	8168
T6	43.51^e^	27.26 to 59.18	1.00	12.22	0.00	1.00	8029
T7	16.10^e^	5.49 to 26.60	1.00	6.26	0.82	1.00	7772
T8	84.78^e^	53.43 to 115.84	1.00	22.05	0.00	1.00	8598
T9	115.91^e^	86.49 to 144.67	1.00	18.39	0.00	1.00	6519
T10	89.47^e^	71.20 to 108.46	1.00	15.77	0.00	1.00	7813
T11	73.07^e^	57.17 to 89.42	1.00	12.98	0.00	1.00	7347
T12	27.69^e^	12.94 to 43.04	1.00	7.56	0.00	1.00	7765
T13	45.99^e^	30.31 to 61.92	1.00	13.01	0.00	1.00	7855
T14	119.30^e^	93.16 to 145.87	1.00	16.15	0.00	1.00	6735
T15	56.74^e^	40.07 to 72.96	1.00	9.77	0.00	1.00	8048
T16	107.86^e^	81.17 to 133.61	1.00	19.39	0.00	1.00	8373
T17	57.72^e^	37.47 to 77.20	1.00	14.88	0.00	1.00	8767
T18	66.72^e^	40.75 to 91.96	1.00	14.01	0.00	1.00	7313
T19	94.56^e^	74.90 to 113.90	1.00	16.98	0.00	1.00	7965
T20	62.54^e^	45.72 to 79.42	1.00	13.31	0.00	1.00	9058
T21	44.44^e^	30.24 to 58.59	1.00	10.88	0.00	1.00	8560
T22	57.24^e^	43.62 to 70.66	1.00	9.38	0.00	1.00	7436
T23	45.24^e^	20.23 to 71.17	1.00	11.94	0.00	1.00	7503
T24	48.20^e^	34.92 to 61.39	1.00	9.34	0.00	1.00	8482
T25	47.93^e^	32.46 to 63.25	1.00	12.21	0.00	1.00	8742
T26	22.51	−66.79 to 112.05	0.70	64.98	84.15	1.00	9231
T27	76.92^e^	59.80 to 94.43	1.00	13.93	0.00	1.00	6829
T28	35.80^e^	21.59 to 49.73	1.00	11.07	0.00	1.00	9366
T29	27.47^e^	5.63 to 49.43	0.99	15.80	13.09	1.00	9333
T30	51.20^e^	37.94 to 64.63	1.00	10.68	0.00	1.00	10,463
T31	61.13^e^	48.35 to 74.01	1.00	11.39	0.00	1.00	8859
T32	84.47^e^	63.97 to 104.78	1.00	13.82	0.00	1.00	8314
T33	72.26^e^	48.83 to 95.74	1.00	15.13	0.00	1.00	7530
T34	59.12^e^	46.46 to 72.07	1.00	11.04	0.00	1.00	8186
T35	50.34^e^	39.48 to 61.15	1.00	9.11	0.00	1.00	9152
T36	45.89^e^	31.88 to 60.45	1.00	10.69	0.00	1.00	8367
T37	79.76^e^	65.18 to 94.36	1.00	12.42	0.00	1.00	8393
T38	96.75^e^	76.15 to 117.53	1.00	15.58	0.00	1.00	6420
T39	97.12^e^	74.12 to 119.88	1.00	14.99	0.00	1.00	7432
T40	21.66^e^	1.58 to 42.28	0.98	10.25	11.05	1.00	7083
T41	83.39^e^	64.82 to 102.49	1.00	13.72	0.00	1.00	7958
T42	67.41^e^	53.03 to 81.99	1.00	11.89	0.00	1.00	8166
T43	40.65	−11.52 to 90.81	0.94	33.52	38.51	1.00	7499
T44	48.21^e^	36.79 to 59.67	1.00	9.10	0.00	1.00	8509
T45	42.05^e^	27.71 to 56.25	1.00	10.78	0.00	1.00	9139
T46	11.97	−4.23 to 28.15	0.93	12.00	50.13	1.00	9021
T47	56.68^e^	42.98 to 70.50	1.00	10.92	0.00	1.00	8050
T48	58.27^e^	46.41 to 70.59	1.00	10.36	0.00	1.00	7819
T49	52.76^e^	40.08 to 65.24	1.00	10.18	0.00	1.00	7410

^a^UI: uncertainty interval.

^b^pd: probability of direction.

^c^ROPE: region of practical equivalence.

^d^ESS: effective sample size.

^e^Statistically significant result.

**Table 4:** Target sample results of individual-level linear models assessing sleep duration associated with sleep quality the day before (all individual-level models included the day of the week as a covariate, but the table only shows model parameters for the main predictor, sleep quality).

Target participant	Slope (median of the posterior distribution)	95% UI^a^	pd^b^	ROPE^c^ (+/−)	% UI in ROPE	R-hat	ESS^d^
T1	−6.18	−20.18 to 7.90	0.81	10.30	72.79	1.00	7331
T2	17.18	−1.23 to 35.01	0.97	13.36	33.68	1.00	7898
T3	4.86	−24.38 to 33.47	0.62	16.12	73.18	1.00	7339
T4	−8.14	−26.68 to 10.37	0.81	10.74	61.51	1.00	7692
T5	−11.59	−25.67 to 2.53	0.95	9.77	38.92	1.00	8057
T6	3.99	−12.52 to 20.73	0.68	12.22	84.16	1.00	8124
T7	1.79	−9.04 to 12.40	0.63	6.26	76.76	1.00	7715
T8	−6.92	−44.90 to 31.06	0.64	22.05	75.05	1.00	8294
T9	−6.64	−43.52 to 30.76	0.63	18.39	68.00	1.00	6542
T10	5.38	−16.89 to 29.41	0.68	15.77	82.23	1.00	8276
T11	11.88	−8.52 to 32.29	0.88	12.98	54.38	1.00	7592
T12	−15.31^e^	−29.24 to −1.46	0.98	7.56	11.92	1.00	6981
T13	1.30	−15.99 to 18.29	0.56	13.01	90.63	1.00	8803
T14	−0.44	−30.03 to 29.90	0.51	16.15	76.59	1.00	7033
T15	6.21	−13.53 to 25.51	0.73	9.77	60.83	1.00	7607
T16	−27.16	−56.68 to 2.91	0.96	19.39	29.00	1.00	7630
T17	−11.26	−30.90 to 8.36	0.87	14.88	64.72	1.00	8548
T18	−5.27	−29.15 to 18.45	0.67	14.01	74.54	1.00	7075
T19	2.84	−20.85 to 25.94	0.59	16.98	87.60	1.00	7754
T20	−15.15	−34.71 to 4.14	0.94	13.31	42.17	1.00	7930
T21	−13.72	−28.15 to 0.59	0.97	10.88	34.11	1.00	8292
T22	−14.97	−31.39 to 2.04	0.96	9.38	24.14	1.00	7859
T23	7.89	−17.17 to 32.62	0.73	11.94	59.32	1.00	7688
T24	−17.72^e^	−31.83 to −3.24	0.99	9.34	10.94	1.00	8332
T25	7.69	−9.26 to 24.56	0.82	12.21	71.23	1.00	7155
T26	−7.54	−80.37 to 64.79	0.58	64.98	96.56	1.00	8465
T27	−7.78	−27.82 to 12.37	0.78	13.93	74.72	1.00	8543
T28	−22.29^e^	−36.43 to −8.12	1.00	11.07	3.94	1.00	7913
T29	−15.76	−37.87 to 6.16	0.92	15.80	50.18	1.00	7036
T30	10.45	−4.82 to 25.99	0.91	10.68	51.31	1.00	6695
T31	2.76	−12.58 to 18.24	0.64	11.39	86.79	1.00	6494
T32	−2.39	−27.37 to 23.67	0.57	13.82	74.03	1.00	7438
T33	−1.18	−27.00 to 24.74	0.54	15.13	79.09	1.00	6429
T34	7.50	−7.80 to 23.05	0.83	11.04	68.41	1.00	8185
T35	2.35	−11.15 to 15.76	0.63	9.11	83.18	1.00	7304
T36	−7.26	−22.38 to 7.85	0.83	10.69	67.71	1.00	7446
T37	−8.99	−27.43 to 9.56	0.83	12.42	65.23	1.00	7425
T38	16.81	−7.39 to 40.61	0.92	15.58	45.69	1.00	7448
T39	14.22	−14.82 to 42.98	0.84	14.99	52.11	1.00	7901
T40	−3.62	−25.53 to 18.36	0.63	10.25	64.99	1.00	7758
T41	−19.81	−43.76 to 4.64	0.94	13.72	30.36	1.00	8205
T42	4.94	−12.29 to 21.91	0.72	11.89	80.34	1.00	6956
T43	6.17	−42.60 to 56.27	0.60	33.52	84.63	1.00	7107
T44	4.48	−7.61 to 16.09	0.76	9.10	78.73	1.00	7931
T45	−10.23	−23.89 to 3.28	0.93	10.78	53.33	1.00	8686
T46	10.73	−4.71 to 26.80	0.91	12.00	56.45	1.00	8086
T47	−20.67^e^	−35.59 to −5.83	1.00	10.92	8.02	1.00	9068
T48	−17.71^e^	−32.53 to −3.60	0.99	10.36	13.20	1.00	8895
T49	4.96	−9.25 to 18.87	0.76	10.18	78.56	1.00	8084

^a^UI: uncertainty interval.

^b^pd: probability of direction.

^c^ROPE: region of practical equivalence.

^d^ESS: effective sample size.

^e^Statistically significant result.

### Higher Subjective Stress Is Associated With Shorter Objective Sleep Duration in Most Target Participants, but the Direction of the Temporal Association Varies

**Figure 6:**
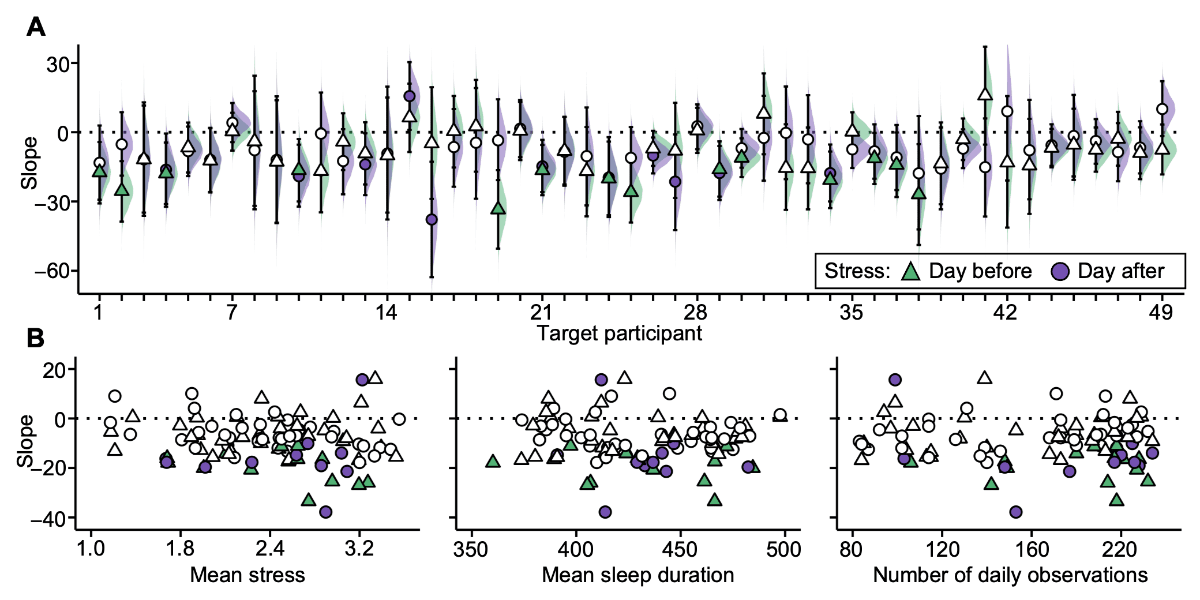
Higher subjective stress associates with shorter objective sleep duration in most individuals. (A) Estimated slopes (corresponding to the median of the posterior distribution) from the individual-level models that assess sleep duration associated with stress the day before (triangles) and with stress the day after (circles) are plotted along the y-axis ordered by participant (x-axis). Statistically significant slope estimates are shaded green (day before) or purple (concurrent). Error bars show 95% uncertainty intervals, and shaded density plots show the full posterior distributions of the slopes. (B) Slope estimates from subpart A are plotted against each participant-level estimate across the study period.

The slope estimate for the association between stress and sleep duration was negative in 86% of all iLMs fitted such that an increase in stress was associated with a decrease in sleep duration (Tables 5 and 6; Figure 6A). This association between stress and sleep duration reached statistical significance in 19 (39%) of the 49 participants (pd>0.975); of these 19 participants, 18 (95%) showed a negative association. Of note, the only participant who showed a significant positive association (T15, for the association between sleep duration and stress the day after) also had no usable actigraphy data over the full spring semester due to a technical issue with their wristband, and their estimated positive slope should therefore be interpreted with caution considering the structured missingness in their data. No other participant in the target sample showed this kind of systematic missingness in either the actigraphy or survey data streams (refer to Figure S1 in Multimedia Appendix 1).

Of the 19 participants who showed a statistically significant relationship between stress and sleep duration, 8 (42%) showed only the *stress-then-sleep* phenotype, that is, days with shorter sleep durations were preceded by higher stress the day before (Figure 6, in green) but not vice versa; 5 (26%) showed only the *sleep-then-stress* phenotype, that is, nights with shorter sleep duration were followed by increased stress the day after (Figure 6, in purple); and 6 (32%) showed bidirectional effects such that nights with shorter sleep duration were preceded by increased stress the day before as well as followed by increased stress the day after. Among participants who showed a significant association between today’s stress levels and sleep duration later that night (14/49, 29%), a 1-point increase in a 5-point perceived stress scale was associated with shorter subsequent sleep duration of a median 17 (range 11-33) minutes across participants. Among participants who showed a significant association between today’s stress levels and last night’s sleep duration (11/49, 22%), a 1-point increase in a 5-point perceived stress scale was associated with shorter previous sleep duration of a median 18 (range 10-38) minutes across participants. These effects would compound to more substantial reductions in sleep duration with greater increases in daily stress.

Individual slope estimates of the association between sleep duration and stress showed no clear pattern of association with individuals’ mean sleep duration or the number of daily observations that went into the model (see Figure 6B for mean sleep duration and number of daily observations). Individuals with higher mean stress tended to have a larger absolute slope estimate (see Figure 6B for mean stress), perhaps because participants with very low stress levels have little variance to be modeled.

**Table 5:** Target sample results of individual-level linear models assessing sleep duration associated with stress the day before (all individual-level models included the day of the week as a covariate, but the table only shows model parameters for the main predictor, stress the day before).

Target participant	Slope (median of the posterior distribution)	95% UI^a^	pd^b^	ROPE^c^ (+/−)	% UI in ROPE	R-hat	ESS^d^
T1	−17.39^e^	−30.84 to −4.23	0.99	9.09	9.64	1.00	7920
T2	−25.47^e^	−38.71 to −12.52	1.00	9.94	0.00	1.00	7532
T3	−11.75	−36.19 to 12.93	0.83	13.68	56.25	1.00	7497
T4	−17.88^e^	−31.13 to −4.48	0.99	8.25	5.45	1.00	7531
T5	−6.59	−17.46 to 4.17	0.89	7.29	55.09	1.00	8189
T6	−12.27	−26.09 to 1.72	0.96	9.98	36.82	1.00	8310
T7	0.37	−7.96 to 8.40	0.53	4.82	78.46	1.00	7568
T8	−3.97	−31.88 to 24.48	0.60	16.89	78.29	1.00	9005
T9	−12.75	−39.38 to 13.84	0.83	14.68	56.53	1.00	6773
T10	−16.42^e^	−29.99 to −2.96	0.99	10.19	16.55	1.00	8429
T11	−16.79	−34.71 to 1.64	0.96	11.91	29.51	1.00	8051
T12	−4.08	−16.42 to 8.22	0.74	6.73	65.81	1.00	7427
T13	−9.18	−22.63 to 4.30	0.91	10.44	57.36	1.00	8887
T14	−10.01	−34.82 to 15.03	0.79	12.84	58.66	1.00	6569
T15	6.40	−8.58 to 21.04	0.80	7.33	54.53	1.00	8457
T16	−4.81	−28.89 to 19.49	0.65	15.46	78.60	1.00	7154
T17	0.47	−15.28 to 15.70	0.52	11.19	88.72	1.00	7480
T18	2.55	−17.09 to 22.65	0.60	11.74	78.35	1.00	8255
T19	−33.47^e^	−50.42 to −16.44	1.00	12.28	0.00	1.00	7885
T20	0.66	−12.06 to 13.07	0.55	8.29	84.76	1.00	7970
T21	−16.43^e^	−27.28 to −5.51	1.00	7.88	3.93	1.00	8275
T22	−8.13	−22.65 to 6.56	0.86	8.08	49.73	1.00	8495
T23	−16.85	−36.46 to 2.27	0.96	9.30	19.97	1.00	6432
T24	−20.04^e^	−35.95 to −4.06	0.99	10.92	11.06	1.00	8168
T25	−25.95^e^	−39.11 to −13.25	1.00	9.44	0.00	1.00	7895
T26	−7.07	−14.59 to 0.61	0.97	5.63	34.56	1.00	8812
T27	−7.98	−28.47 to 12.74	0.78	14.84	76.27	1.00	8484
T28	0.77	−8.97 to 10.58	0.56	7.28	89.44	1.00	8063
T29	−16.06^e^	−27.87 to −3.96	1.00	8.52	8.74	1.00	7850
T30	−11.06^e^	−19.45 to −2.63	1.00	5.92	9.27	1.00	8544
T31	7.99	−9.54 to 25.47	0.81	13.61	74.60	1.00	8993
T32	−15.49	−33.63 to 3.45	0.95	10.88	30.23	1.00	9197
T33	−15.60	−33.42 to 2.04	0.96	10.69	28.00	1.00	7358
T34	−20.70^e^	−32.87 to −8.37	1.00	9.07	0.79	1.00	7973
T35	0.24	−7.97 to 8.63	0.52	5.75	87.05	1.00	8332
T36	−11.30^e^	−22.46 to −0.28	0.98	7.70	25.16	1.00	6861
T37	−14.13^e^	−28.07 to −0.10	0.98	9.32	23.84	1.00	7889
T38	−26.90^e^	−48.84 to −5.17	0.99	15.98	14.28	1.00	7520
T39	−13.52	−31.21 to 4.22	0.93	9.20	29.79	1.00	7380
T40	−3.02	−12.06 to 5.95	0.75	4.39	59.48	1.00	7465
T41	15.91	−5.36 to 37.06	0.93	12.45	35.81	1.00	7703
T42	−13.10	−41.31 to 15.65	0.82	20.77	71.60	1.00	8825
T43	−14.40	−35.39 to 5.80	0.92	14.06	48.56	1.00	7357
T44	−6.76	−15.22 to 1.43	0.94	6.47	46.99	1.00	8141
T45	−5.39	−20.66 to 10.17	0.75	12.13	81.79	1.00	8410
T46	−7.56	−17.10 to 2.05	0.94	6.98	44.73	1.00	7513
T47	−2.84	−14.49 to 8.84	0.69	8.30	83.36	1.00	8012
T48	−9.00	−20.33 to 2.12	0.95	7.59	39.17	1.00	8326
T49	−7.55	−18.29 to 3.36	0.92	7.85	52.52	1.00	7613

^a^UI: uncertainty interval.

^b^pd: probability of direction.

^c^ROPE: region of practical equivalence.

^d^ESS: effective sample size.

^e^Statistically significant result.

**Table 6:** Target sample results of individual-level linear models assessing sleep duration associated with stress the day after (all individual-level models included the day of the week as a covariate, but the table only shows model parameters for the main predictor, stress the day after).

Target participant	Slope (median of the posterior distribution)	95% UI^a^	pd^b^	ROPE^c^ (+/−)	% UI in ROPE	R-hat	ESS^d^
T1	−13.23	−29.23 to 2.91	0.95	9.09	29.61	1.00	5797
T2	−5.24	−18.80 to 8.68	0.77	9.94	76.74	1.00	8875
T3	−11.77	−34.81 to 11.56	0.85	13.68	56.47	1.00	7338
T4	−16.22^e^	−32.32 to −0.57	0.98	8.25	15.11	1.00	8021
T5	−8.21	−18.99 to 2.43	0.94	7.29	42.56	1.00	7377
T6	−11.94	−25.88 to 1.90	0.96	9.98	37.93	1.00	8926
T7	4.12	−4.28 to 12.64	0.84	4.82	56.73	1.00	8095
T8	−7.79	−33.38 to 17.75	0.73	16.89	77.04	1.00	8068
T9	−11.98	−39.16 to 15.54	0.80	14.68	57.80	1.00	8224
T10	−19.06^e^	−32.23 to −5.39	1.00	10.19	7.72	1.00	7881
T11	−0.62	−18.60 to 17.18	0.53	11.91	85.57	1.00	7375
T12	−12.49	−26.89 to 1.31	0.96	6.73	18.85	1.00	7661
T13	−13.95^e^	−27.24 to −0.47	0.98	10.44	30.02	1.00	7455
T14	−9.24	−37.81 to 19.76	0.73	12.84	55.80	1.00	5908
T15	15.64^e^	1.01 to 30.35	0.98	7.33	11.11	1.00	7561
T16	−37.82^e^	−62.78 to −12.78	1.00	15.46	1.60	1.00	7345
T17	−6.43	−23.61 to 10.03	0.78	11.19	72.94	1.00	7712
T18	−4.56	−28.81 to 19.13	0.65	11.74	66.61	1.00	8545
T19	−3.52	−20.73 to 14.26	0.66	12.28	83.96	1.00	8844
T20	1.55	−10.79 to 13.91	0.60	8.29	84.36	1.00	7304
T21	−14.76^e^	−25.94 to −3.31	0.99	7.88	9.48	1.00	7913
T22	−8.48	−23.16 to 6.68	0.87	8.08	47.68	1.00	8106
T23	−10.36	−31.70 to 10.72	0.83	9.30	44.67	1.00	7312
T24	−19.63^e^	−36.73 to −2.13	0.99	10.92	14.05	1.00	7158
T25	−11.10	−24.15 to 2.27	0.95	9.44	39.41	1.00	8495
T26	−10.11^e^	−17.83 to −2.28	1.00	5.63	10.24	1.00	8767
T27	−21.40^e^	−42.43 to −0.97	0.98	14.84	25.22	1.00	8373
T28	2.60	−7.35 to 12.04	0.70	7.28	84.95	1.00	8551
T29	−17.68^e^	−29.37 to −5.97	1.00	8.52	3.66	1.00	8521
T30	−6.95	−15.37 to 1.40	0.95	5.92	40.05	1.00	8895
T31	−2.47	−20.99 to 15.76	0.60	13.61	88.88	1.00	9079
T32	−0.28	−20.33 to 19.80	0.51	10.88	76.02	1.00	8377
T33	−3.11	−22.10 to 16.05	0.62	10.69	73.59	1.00	7235
T34	−17.71^e^	−29.99 to −5.33	1.00	9.07	6.43	1.00	8398
T35	−7.43	−15.50 to 0.75	0.96	5.75	32.79	1.00	8137
T36	−8.27	−20.14 to 3.43	0.92	7.70	45.84	1.00	6671
T37	−10.86	−25.05 to 3.13	0.94	9.32	41.21	1.00	7458
T38	−17.78	−42.09 to 7.02	0.93	15.98	43.95	1.00	8022
T39	−15.79	−33.41 to 2.06	0.96	9.20	21.49	1.00	7141
T40	−7.11	−15.56 to 1.26	0.95	4.39	24.88	1.00	7108
T41	−15.15	−36.52 to 5.96	0.92	12.45	39.48	1.00	8726
T42	9.05	−20.92 to 38.93	0.72	20.77	79.25	1.00	8862
T43	−7.80	−29.05 to 14.03	0.76	14.06	72.40	1.00	7545
T44	−5.63	−14.57 to 3.44	0.89	6.47	57.66	1.00	8151
T45	−1.63	−19.16 to 16.16	0.58	12.13	86.41	1.00	8594
T46	−3.81	−13.64 to 5.68	0.79	6.98	75.80	1.00	8400
T47	−8.61	−21.25 to 3.95	0.91	8.30	47.73	1.00	7590
T48	−6.65	−17.83 to 4.77	0.87	7.59	56.89	1.00	7442
T49	10.06	−1.66 to 22.09	0.96	7.85	34.47	1.00	8521

^a^UI: uncertainty interval.

^b^pd: probability of direction.

^c^ROPE: region of practical equivalence.

^d^ESS: effective sample size.

^e^Statistically significant result.

### Person-Specific Estimates Get Attenuated in Group-Based Modeling

**Figure 7:**
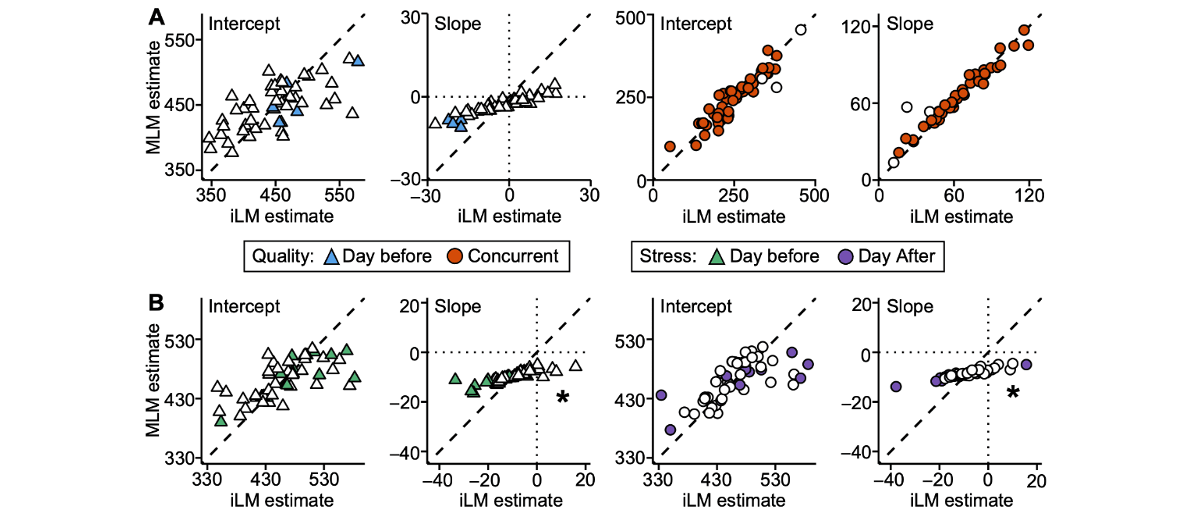
Estimates of the intercepts and slopes from the individual-level linear models (iLMs; x-axis) for each participant are compared to the random effects estimated from a group-based multilevel linear model (MLM; y-axis) and presented separately for (A) associations between sleep duration and sleep quality and (B) associations between stress and sleep duration (bottom row). Symbol shading signifies statistically significant slopes in the iLMs. Dashed diagonal lines represent identical estimates between the individual- and group-based approaches. *Attenuation of individual estimates in the MLM compared to the iLMs.

A comparison of the estimates obtained through the iLM and those of an MLM demonstrates that individual-level estimates get systematically attenuated in a group-based approach when there is between-person heterogeneity in the effects (Figure 7). When there was a strong effect and small between-person variability in the tested associations, as with the association between sleep duration and concurrent sleep quality, the iLMs provided slope estimates nearly identical to those estimated through an MLM (Figure 7A). However, for associations that showed a weaker effect and greater degree of between-person variability, group-level approaches systematically flatten individually tailored effect sizes or even reverse the sign of the association (Figure 7B). We observed this in the lead-lag associations between stress and sleep duration (as highlighted with asterisks in Figure 7) and to some degree in the lagged association between sleep quality and sleep duration.

### Examining Raw Within-Individual Data Informs the iLM Results

**Figure 8:**
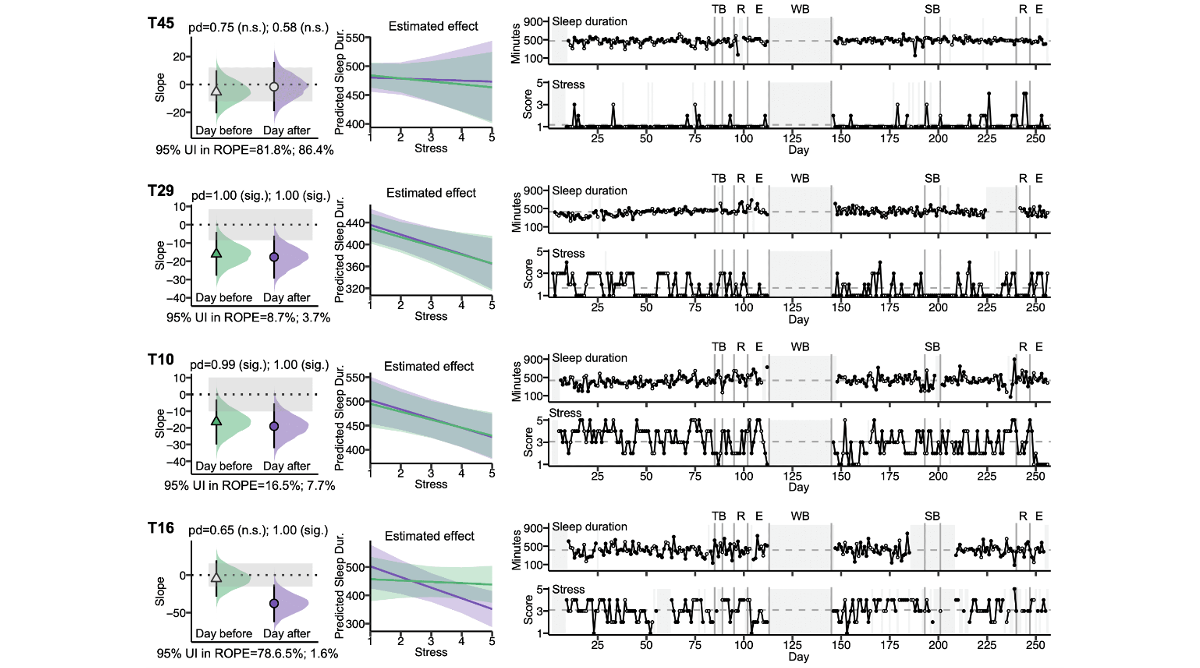
Intensive within-individual longitudinal data reveal differences among individuals. Results are shown for individual-level models testing the association between sleep duration and stress the day before (green) and between sleep duration and stress the day after (purple). Column 1 shows the models’ estimated slopes (computed as the median of the posterior distribution) and uncertainty metrics. Symbol shading signifies statistically significant slopes. Error bars show 95% uncertainty intervals (UIs). Shaded density plots show the full posterior distributions of the slopes; gray shaded areas show the regions of practical equivalence (ROPEs). Column 2 shows the models’ predicted sleep duration as a function of stress; shading around the lines indicates 95% UIs. The time series panels in column 3 show daily observations of actigraphy-derived sleep duration and survey-based perceived stress. Gray dashed lines show the participant’s mean value across the study period. Vertical lines indicate landmark events (labeled) in the academic calendar. Gray shading indicates missing data during the school terms or the winter break, which were excluded from the individual-level linear models. Dur.: duration; E: examinations period; n.s.: not statistically significant; pd: probability of direction; R: reading period; SB: spring break; sig.: statistically significant; TB: Thanksgiving break; WB: winter break.

A closer look at individual participants’ data reveals important considerations for the application and interpretation of the iLMs (Figure 8). As with other statistical models, the iLM is unlikely to detect stable associations for participants with too little variability in their data; for instance, participant T45 had consistently low stress levels over the course of the year, with occasional, small increases in stress tied to periods with increased academic demands (eg, ahead of midterm and final examinations periods). This participant’s individual linear models showed null results for both directions of stress-sleep associations (pd<0.975). Meanwhile, participant T29 also had low baseline stress levels, but they presented more frequent and substantial rises in stress throughout the year. Participant T29’s iLMs showed bidirectional negative associations between stress and sleep duration (pd>0.975).

While variability in daily observations over time is necessary to detect linear associations within the iLM, variability alone is not sufficient. Participant T10 showed substantial fluctuations in stress and sleep duration throughout the year, and their iLMs found bidirectional negative associations between stress and sleep duration (pds>0.975). Meanwhile, participant T16 also presented substantial variability in stress and sleep duration, and while their iLM detected a significant association between sleep duration and stress the day after (pd=1.00), no association was detected in the opposite direction (pd=0.65).

## Discussion

### Principal Findings

Stress levels and sleep duration interact in an individual’s daily life, but research has yielded mixed findings regarding the temporal directionality of their associations, with changes in stress preceding changes in sleep, changes in sleep preceding changes in stress, or both. Here, we leveraged a novel individual-level linear regression modeling iLM framework to obtain precision estimates of day-to-day associations between self-reported stress levels and actigraphy-derived sleep duration in a sample of first-year college students studied continuously for a full academic year. While most of the participants (45/49, 92%) showed a negative association between daily stress levels and sleep duration, the temporal direction of the association varied, with all types of lead-lag association previously reported at the group level present within distinct individuals in our sample.

In agreement with prior literature, our results within individuals confirm that stress levels and sleep duration are closely and inversely related in daily life [10-19]. Nearly four-tenths (19/49, 39%) of the participants—each considered an independent test of the association—showed a statistically significant effect (in either temporal direction), revealing that day-to-day changes in stress levels or sleep duration can reliably predict one another in the real world in a substantial portion of the sample. The slope estimate for the association between stress and sleep duration was negative in 86% of the fitted models. Within the estimated slopes that reached statistical significance, all but one were negative. This suggests that, for most individuals, increased stress levels are associated with shorter rather than longer sleep duration in the surrounding days, consistent with findings showing that periods of heightened stress levels coincide with periods of reduced sleep [11-14]. Critically, we provide a framework to obtain individually tailored estimates of these day-to-day associations. The individual-level slope estimates ranged from 10 to 38 minutes in shorter sleep duration per 1-unit increase in a 5-point perceived stress scale, suggesting that a change in stress levels can be associated with substantial changes in sleep duration, especially when daily stress levels increased by several units. It should be noted that while negative associations between sleep duration and stress levels predominated in our sample, it is possible that some individuals in the wider population show positive associations but were not captured in our study.

Our precision approach further revealed that the temporal directionality of the association between stress and sleep duration varied from person to person, representing all patterns of results reported by prior, group-level studies. For some of the participants (8/49, 16%), heightened stress during the day associated with shorter sleep later that night but not vice versa, in agreement with group results reported in the studies by Marcusson-Clavertz et al [15] and Slavish et al [16]; for others (5/49, 10%), shorter sleep associated with heightened stress the next day but not vice versa, in agreement with group results in the study by Sin et al [17]; and yet others (6/49, 12%) showed both directions of association, in agreement with group results in the studies by Doane and Thurston [18] and Yap et al [19]. Daily psychological and behavioral experiences such as perceived stress and sleep duration thus seem to interact in person-specific ways, rather than being uniform across the population.

For individuals showing the *stress–then–reduced-sleep* phenotype, experiences of heightened stress (eg, due to an impending final examination or social conflict) might elicit hyperarousal and rumination [13,51-53], as well as behaviors aimed at mitigating the source of stress (eg, studying or socializing with friends late into the night), all of which can delay sleep and reduce its overall duration. Moreover, for those showing the *reduced-sleep–then–stress* phenotype, shortened sleep durations might enhance their sensitivity to (and undermine their ability to cope with) academic, interpersonal, or other stressors and thus make them more likely to experience heightened stress levels [10,54,55]. For some individuals, both patterns of effect might occur, with changes in stress levels and sleep duration reinforcing one another and resulting in chains of days with heightened stress and nights of short sleep that succeed one another.

Critically, our results suggest that group-level studies might report inconsistent findings, at least partly, because the dynamic interaction between daily stress levels and sleep duration varies from person to person. When data are aggregated at the group level, individual phenotypes might be obscured, and the resulting group-level estimates are suggestive of generalized effects when in fact they might only apply to a fraction of the sample. Even when hierarchical group models allow for fitting individual estimates, these are biased by the group and tend to attenuate (or *shrink*) the estimation of individual effects [26]. A comparison of individual slope estimates of stress-sleep associations derived from our iLMs and those derived from an MLM with random intercepts and slopes starkly demonstrated this group bias: the group MLM estimated individual slopes that were systematically attenuated or even reversed sign compared to those estimated by our iLMs.

These results demonstrate the utility of individual-level modeling for characterizing real-world behavioral and psychological dynamics [27-29,34]. Our approach leverages mobile and wearable technology and provides a fit-for-purpose methodology that can turn these devices’ large-scale longitudinal measurements into meaningful insights. The iLM’s model specification is parsimonious by design and easy to interpret, including a single linear term for the main predictor of interest and a day-of-the-week covariate to account for the weekly structure in the outcome variable. Diagnostic checks confirmed that this simple model specification was powerful enough to capture real-world, stable linear associations between psychological phenomena and objective behaviors, while accounting for the time-related dependencies in the daily observations. Individual tailoring is achieved by fitting only 1 person’s data within a model, but the model specification remains identical across individuals, allowing for direct between-person comparisons of results, including estimating the relative prevalence of different phenotypes in the group.

The iLM framework is readily applied to a single individual’s data, making it useful for multiple real-world purposes beyond fundamental research that are increasingly gaining interest in the fields of consumer health informatics and digital health [21,31,32]. The iLM can be applied to data collected through personal devices for self-monitoring as well as for precision approaches in health care settings, where clinicians might use a patient’s data to triage intervention plans. Taking these results as an example, stress management interventions might be first prioritized among individuals for whom heightened stress precedes shortened sleep, while sleep interventions might be prioritized among individuals for whom shortened sleep precedes heightened stress. Moreover, this work might inform a growing body of research and products combining multiple data streams from wearables and smartphones along with machine learning techniques to *predict* experiences of stress [56,57]. Although prediction was not the focus of our work, understanding the person-specific association between stress and sleep duration, as well as the weekly behavioral patterns revealed by our actigraphy and survey data and individualized approach, could potentially contribute to the identification of periods when individuals are more likely to experience stress so that timely interventions can be offered.

The iLM offers a simple yet powerful precision framework for the estimation of real-world psychological and behavioral associations within the individual, but the results should be interpreted carefully in light of its assumptions and limitations. First, the iLM’s assumptions of linear, stable associations between the predictor and outcome variables are a deliberate attempt to simplify real-world behavioral and psychological dynamics that are highly complex. In the context of this study, these features allowed us to estimate college students’ day-to-day stress-sleep associations that are stable across the fluctuating demands on students within the school semesters as well as straightforward to interpret. However, it is possible that the association between stress and sleep duration is context dependent, varying as a function of the specific source of stress experienced by the individual (eg, academic vs interpersonal) and the broader seasonal demands (eg, whether school is in session). In fact, given the possibility of the latter, we decided a priori to exclude data collected during the winter break from our models. Future research could examine how these contextual demands influence stress-sleep associations, as well as explore nonlinear associations or cumulative effects over time.

Our analyses leveraged leading and lagging patterns in each individual’s time series of stress and sleep duration measures to ascertain the temporal directionality of their association (eg, stress during the day as a predictor of subsequent sleep duration later that night), but we did not implement a controlled experimental manipulation and cannot establish a causal relationship. While our use of intensive longitudinal data collected in the real world grants our results ecological validity, it also exposes them to confounders; for example, it is possible that a significant association between short sleep and higher stress the next day could be explained by the anticipated demands of the next day, such as an examination. Rather than short sleep duration *causing* higher stress the next day, the examination might be the primary cause behind both the short sleep (staying up late to prepare) and the stress reported the next day (heightened stress during test taking).

While our current intensive longitudinal data set and individual-level modeling framework passed sanity checks and diagnostics that confirmed data quality and adequate model specification, researchers applying our framework to other data sets and research questions should scrutinize the appropriateness of the data and the model before interpreting the results. Mobile and wearable technologies enable the collection of large behavioral data sets over time, but quantity does not guarantee quality, and long study periods require extra vigilance to ensure that participants remain compliant over time. Quality checks should confirm that the data collected are capturing expected real-world behavior (eg, as suggested by the intuitive changes we observed in students’ behavior between school terms and breaks as well as between weekdays and weekends). Even if the available data are substantial, and participant compliance is high, small variability in the metrics under study could still impede obtaining meaningful estimates of their associations, as demonstrated by participants with minimal fluctuations in stress levels over the course of the academic year. Moreover, investigators should be careful to identify appropriate uses, sanity checks, and interpretations of the iLM for their population of study; for example, part of our iLM validation process included testing the expected positive association between objective sleep duration (measured via the actigraphy wristband) and the participant’s subjective rating for the same sleep event (reported via a daily smartphone survey). While we expect that generally healthy participants will tend to rate nights of shorter-than-usual sleep duration as lower quality, it might not always be advisable to assume a simple linear association between objective sleep duration and subjective sleep quality, especially when modeling data from patients with sleep and psychiatric disorders.

### Conclusions

Our novel iLM framework leveraged intensive longitudinal data from mobile and wearable devices to obtain individually tailored estimates of day-to-day associations between subjective stress levels and objective sleep duration. While stress and sleep duration were inversely related in most of the participants (45/49, 92%), the iLM revealed that the temporal direction of these associations is person specific, identifying a variety of individual phenotypes that may account for the diverse group-level findings reported in prior literature. Our results demonstrate the utility of individual-level modeling approaches for the assessment of behavioral and psychological associations in the real world. An individualized approach offers a foundation for the characterization of life dynamics at both the individual and group levels, as well as for the development of precision health and well-being interventions tailored to the individual.

## Appendix

Multimedia Appendix 1 Additional figures show participant-level missing data, comparison between Bayesian and frequentist individual-level linear model estimates, and group-level sleep and stress metrics in the target sample.

## Abbreviations

DPSleep: deep phenotyping of sleep
ESS: effective sample size
iLM: individual-level linear model
MCMC: Markov chain Monte Carlo
MLM: multilevel linear model
pd: probability of direction
ROPE: region of practical equivalence
UI: uncertainty interval

## Data Availability

The data sets generated during this study are not publicly available due to concerns related to participant identifiability but may be available from the corresponding author on reasonable request.
