# Supplementary material for: Precision Assessment of Real-World Associations Between Stress and Sleep Duration Using Actigraphy Data Collected Continuously for an Academic Year: Individual-Level Modeling Study
Source: JMIR Form Res. 2024 Apr 30;8:e53441. doi: 10.2196/53441 (PMC11094608; doi:10.2196/53441)
Supplement: Multimedia Appendix 1 [file formative_v8i1e53441_app1.docx]

**MultimediaAppendix 1: Additional figures show participant-level missing data, comparison between Bayesian and frequentist individual-level linear model estimates, and group-level sleep and stress metrics in the target sample.**

**
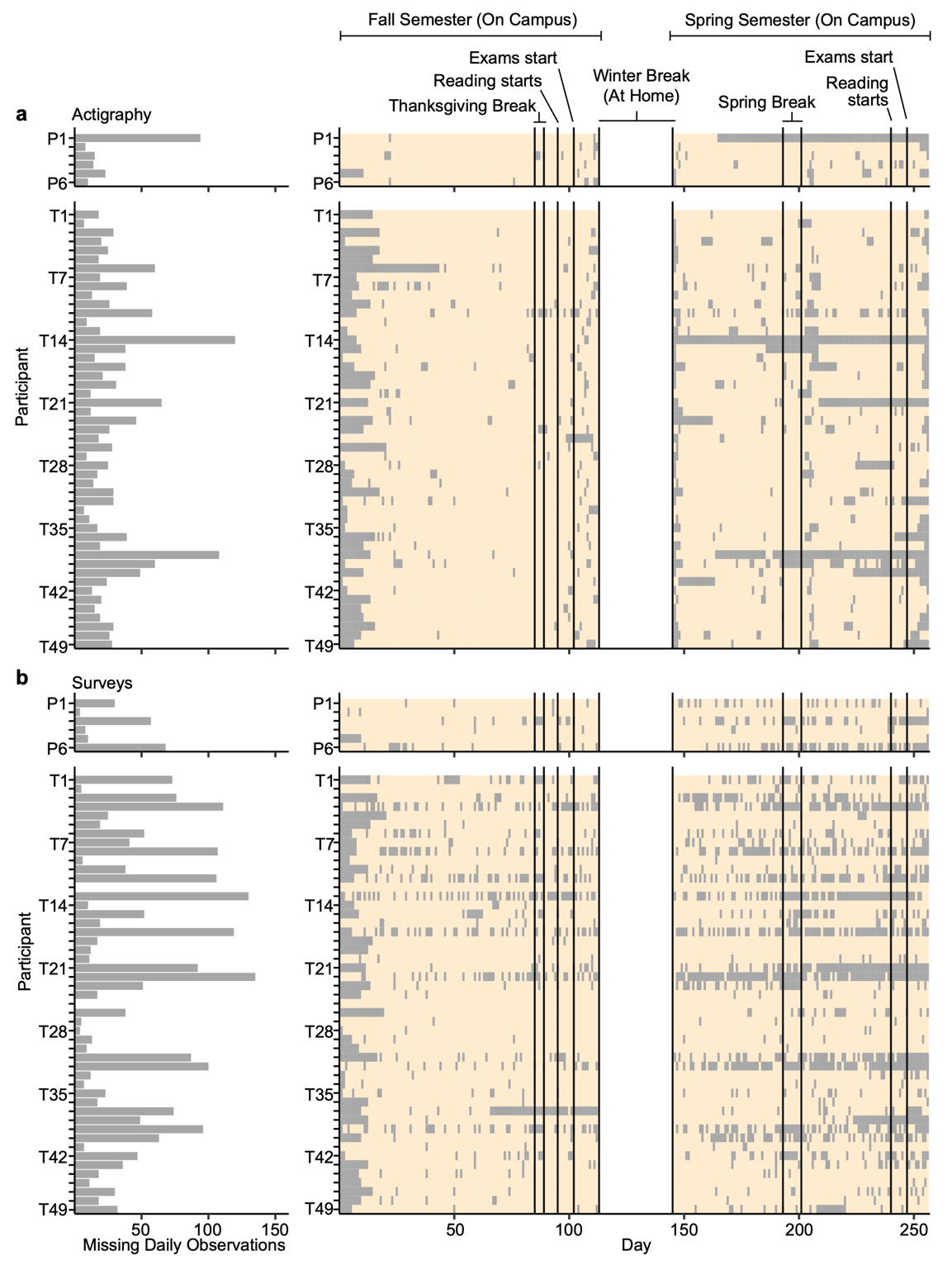
**

**Figure S1. Missing daily observations per participant in actigraphy (a) and survey data (b).** The left-hand panels show missing daily observations per participant (out of a total possible of 223, which excludes data collected during Winter Break). The right-hand panels show daily observations over the study period, colored by whether they were available (cream color) or missing (grey color). Landmark events in the academic calendar are indicated by vertical black lines, labeled at the top. Participants in the Pilot dataset are labeled with a “P”, and participants in the Target dataset are labeled with a “T”.


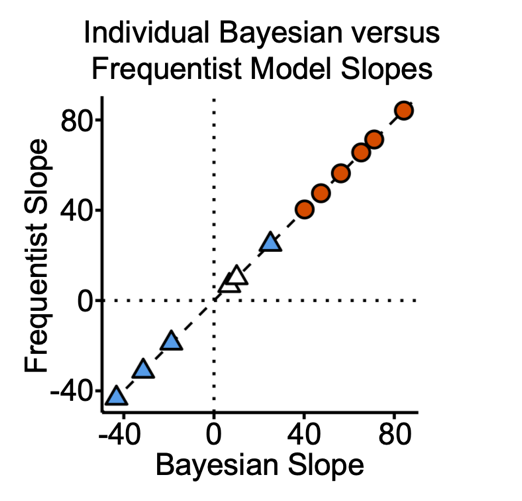


**Figure S2.** **Slope estimates from Bayesian individual-level models were virtually the same as those obtained with identically specified models in a frequentist inference framework.** The dashed diagonal line represents identical values between the Bayesian and Frequentist approaches. Triangles represent the slope estimates from the models assessing Sleep Duration associated with Sleep Quality the Day Before, while Circles represent slope estimates from the models assessing Sleep Duration associated with Concurrent Sleep Quality. Symbol shading signifies statistically significant slopes.


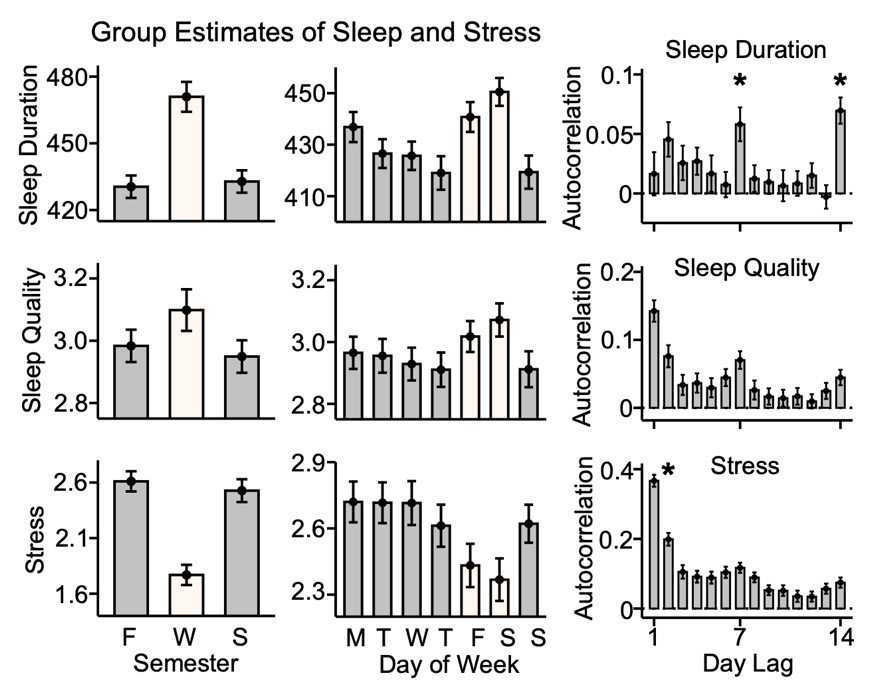


**Figure S3. Group-aggregated Sleep and Stress metrics in target dataset recapitulate the structure of the academic calendar seen in pilot sample.** Target participants’ group (between-person) means of Sleep Duration (in minutes, top row), Sleep Quality (5-point Likert scale, middle row), and perceived Stress (5-point Likert scale, bottom row), aggregated by school semester (Fall and Spring) and Winter Break (first column) and by day of the week (second column). The third column shows between-person means of autocorrelation estimates over a 14-day window. Asterisks highlight that autocorrelations were strongest at a 7-day lag (and again at 14-day lag) for Sleep Duration and at 1-day and 2-day lags for perceived Stress. Error bars show standard error of the mean. F = Fall semester, W = Winter break, S = Spring semester; M = Monday, T = Tuesday, W = Wednesday, T = Thursday, F = Friday, S = Saturday, S = Sunday.
